# Supplementary material for: Public Baseline and shared response structures support the theory of antibody repertoire functional commonality
Source: PLoS Comput Biol. 2021 Mar 1;17(3):e1008781. doi: 10.1371/journal.pcbi.1008781 (PMC7951972; doi:10.1371/journal.pcbi.1008781)
Supplement: S1 Text — A description of the methodology used to benchmark new ESS thresholds for use on repertoire data, and for evaluating a set of 20 important interface residues for orientation template assignment. (PDF) [file pcbi.1008781.s001.pdf]

Supporting Information Text for:
'Public Baseline and Shared Response Structures Support the Theory of Antibody Repertoire Functional Commonality'

Matthew I. J. Raybould, Claire Marks, Aleksandr Kovaltsuk, Alan P. Lewis, Jiye Shi, Charlotte M. Deane

Immunoglobulin Gene Sequencing (Ig-seq) Environment-Specific Substitution Scores

The original antibody Environment-Specific Substitution Score (ESS) threshold used by FREAD (25 for all loops, and loop lengths) was derived by Choi and Deane [1,2] based on benchmarking of performance on the Protein Data Bank (PDB). This involved first excising the CDRH3 loop from known PDB antibody structures, and then feeding the framework structure and excised CDRH3 loop sequence into FREAD, asking it to predict the next-best (non-self) PDB CDRH3 template for modelling, and measuring the achieved root-mean-squared deviation (RMSD). However, the CDRH3 loops in Ig-seq samples of natural antibody repertoires deviate significantly from the heavily engineered PDB, which has high redundancy and many closely related structures that help to improve mean performance. To show this, we calculated the typical ESS value of the top-ranked FREAD CDRH3 template for an Ig-seq study of natural antibodies (Fig. S1A), comparing it to the ESS of the top-ranked FREAD CDRH3 template obtained when modelling-in PDB CDRH3 loops (Fig. S1B, blinding access to the same structure as a template). The highest ranked template has the lowest anchor residue C_α RMSD, after surpassing the baseline threshold of ESS 25. The Ig-seq data benefits from far fewer high ESS scores, so application of the original thresholds on Ig-seq data would be expected to achieve below-headline performance. To both estimate our true performance on Ig-seq data and derive new CDRH3 loop ESS thresholds more appropriate for Ig-seq data, we subsampled the PDB comparison set to match the top-ranked ESS distributions seen for each length bin in the Ig-seq sample. Based on this sample, we predict that we should achieve a good accuracy (mean RMSD of 2.54Å) with acceptable coverage on Ig-seq data using the following CDRH3 ESS cutoffs: Lengths 5-8, ESS ≥ 25; Lengths 9-10, ESS ≥ 35; Lengths 11+, ESS ≥40.

Determining Interface Residues Key for Orientation Prediction

The 1,129 sequence non-redundant Fvs with resolution ≤ 2.5Å were taken from the SAbDab database [3] (12th February 2019), and all residues found to lie in the VH-VL interface were identified. This was achieved by first calculating the relative solvent accessible surface area ($SASA_{rel}$, Shrake-Rupley Algorithm [4]) to determine the absolute SASA of each residue and dividing this number by the theoretical maximum SASA for that residue. The $SASA_{rel}$ of each position in the complex was then compared to the value for the equivalent position in the separated VH and VL chains (coordinates of the partner chain deleted). The resulting 52 residue positions (Table S1), which appeared in at least 80\% of complexes, and whose $SASA_{rel}$ was reduced by an average of at least 5% in at least 10% of those complexes, were taken forward. To reduce this further, we performed a Random Forest regression analysis [standard scikit-learn implementation, 500 estimators] over these 52 residues to the 6 ABangle parameters [5] that have been shown to characterise VH-VL orientation. Firstly, we confirmed that the 1129 interfaces constituted a representative sample of all ABangle parameter space — essential to learn genuine residue to ABangle parameter responses. Each interface was then flattened and one-hot-encoded (21 columns for each position, for the 20 natural amino acids or a deletion/missing residue) to yield a 1129x1092 matrix which was separately regressed against each ABangle parameter (6 x (1129x1) vectors). Out-of-bag validation was used to estimate R^2 values, and showed predictive performance ranging from

an estimated R^2 of 0.35-0.54. We calculated feature importance, and derived a new one-hot-encoded interface for each complex using only the 20 positions (Table S1) that were present in the top-5 most important features across the 6 parameters. Predicted performance dropped by an average estimated R^2 of only 0.05 across the six parameters (min: 0, max: -0.11).

The coverage and accuracy of the 52 and 20 residue interface definitions at predicting orientation RMSD was also assessed. Orientation RMSD between two complexes was measured by first aligning their VH domains and measuring the C_α distances between common VL positions, then by aligning their VL domains and measuring the C_α distances between common VH positions, and finally dividing by two. A 'correct/incorrect' orientation threshold RMSD of 1.5Å was chosen by measuring the variation in pairwise orientation RMSD observed for sequence-identical SAbDab complexes, *i.e*. an estimate for the experimental limitation on what constitutes a `correct' orientation RMSD. A threshold of 1.5Å captures 92% of sequence identical Fvs (Fig. S2). An orientation sequence identity threshold was then chosen for the 20-residue and 52-residue interface definitions that balanced acceptable coverage with a high proportion of Fvs within 1.5Å above the threshold (Fig. S3). We chose 82% for the 52-residue definition, and 85% for the 20-residue definition. Coverage was comparable and accuracy only slightly reduced (80.2% to 77.8%) on narrowing the interface definition to 20 residues.

References

1. Yoonjoo Choi and Charlotte M. Deane. Accurate loop structure prediction using a database search algorithm. *Proteins*, 78(6):1431—1440, 2010. doi: 10.1002/prot.22658.
2. Yoonjoo Choi and Charlotte M. Deane. Predicting antibody complementarity determining region structures without classification. *Mol. BioSyst.*, 7(12):3327—3334, 2011. doi: 10.1039/C1MB05223C.
3. James Dunbar, Konrad Krawczyk, Jinwoo Leem, Terry Baker, Angelika Fuchs, Guy Georges, Jiye Shi, and Charlotte M. Deane. SAbDab: the Structural Antibody Database. *Nucleic Acid Res.*, 42(D1):D1140—D1146, 2014. doi: 10.1093/nar/gkt1043.
4. Andrew Shrake and John A. Rupley. Environment and exposure to solvent of protein atoms. Lysozyme and insulin. *J. Mol. Biol.*, 79(2):351—364, 1973. doi: 10.1016/0022-2836(73)90011-9.
5. James Dunbar, Angelika Fuchs, Jiye Shi, and Charlotte M. Deane. ABangle: characterising the VH-VL orientation in antibodies. *Protein Eng. Des. Sel.*, 26(10):611—620, 2013. doi: 10.1093/protein/gzt020.
