# Supplementary figures and images for: Public Baseline and shared response structures support the theory of antibody repertoire functional commonality
Source: PLoS Comput Biol. 2021 Mar 1;17(3):e1008781. doi: 10.1371/journal.pcbi.1008781 (PMC7951972; doi:10.1371/journal.pcbi.1008781)

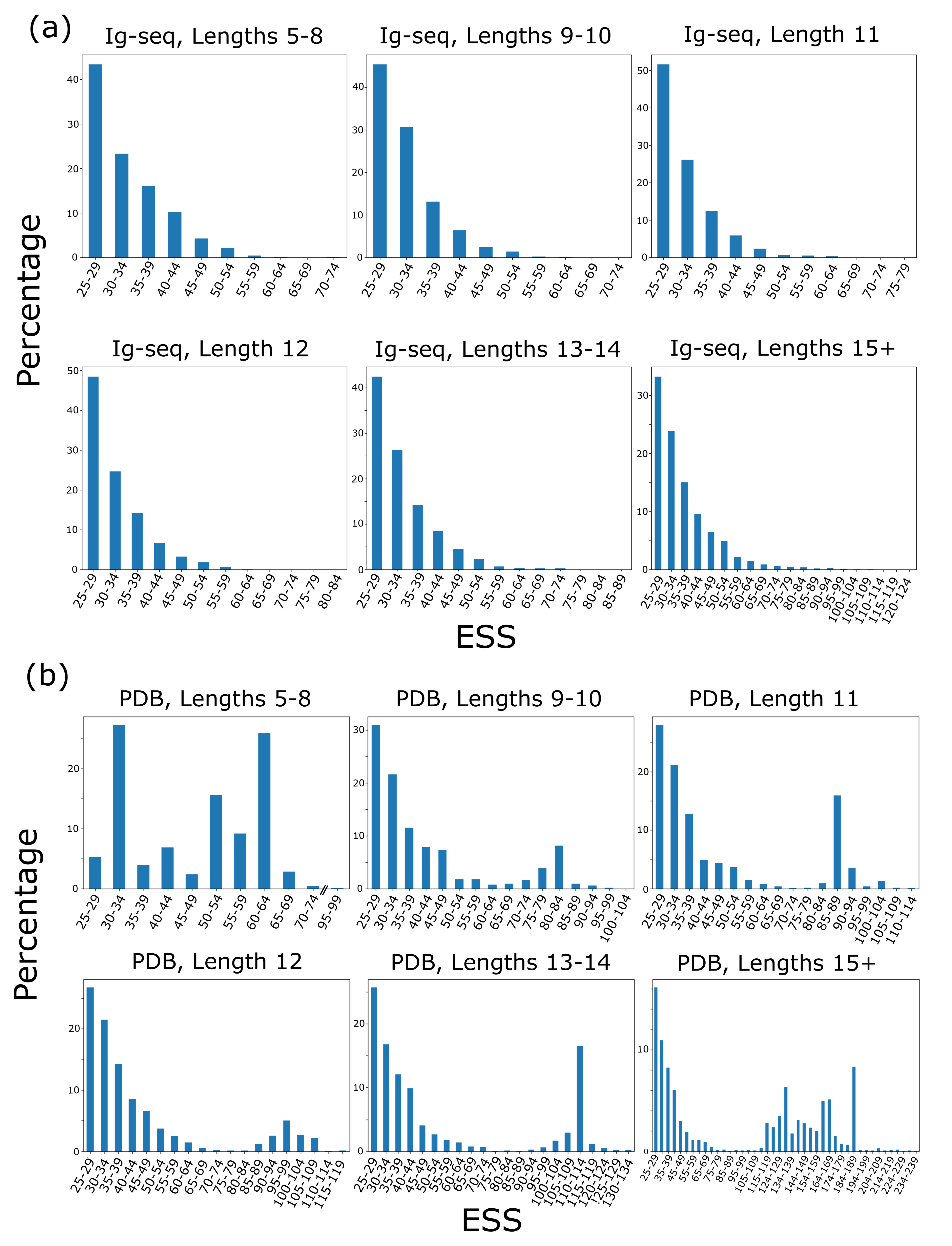

Supplement: S1 Fig — The percentage of each FREAD top-ranked CDRH3 templates with an Environment Specific Substitution Score (ESS) within the labelled bin for (a) a typical Ig-seq dataset, and (b) the Protein Data Bank (blinded to self). The two sets have very different distributions; notably Ig-seq datasets rarely contain CDRH3 loops with extremely high ESS scores to dataset templates. (PNG) [file pcbi.1008781.s002.png]

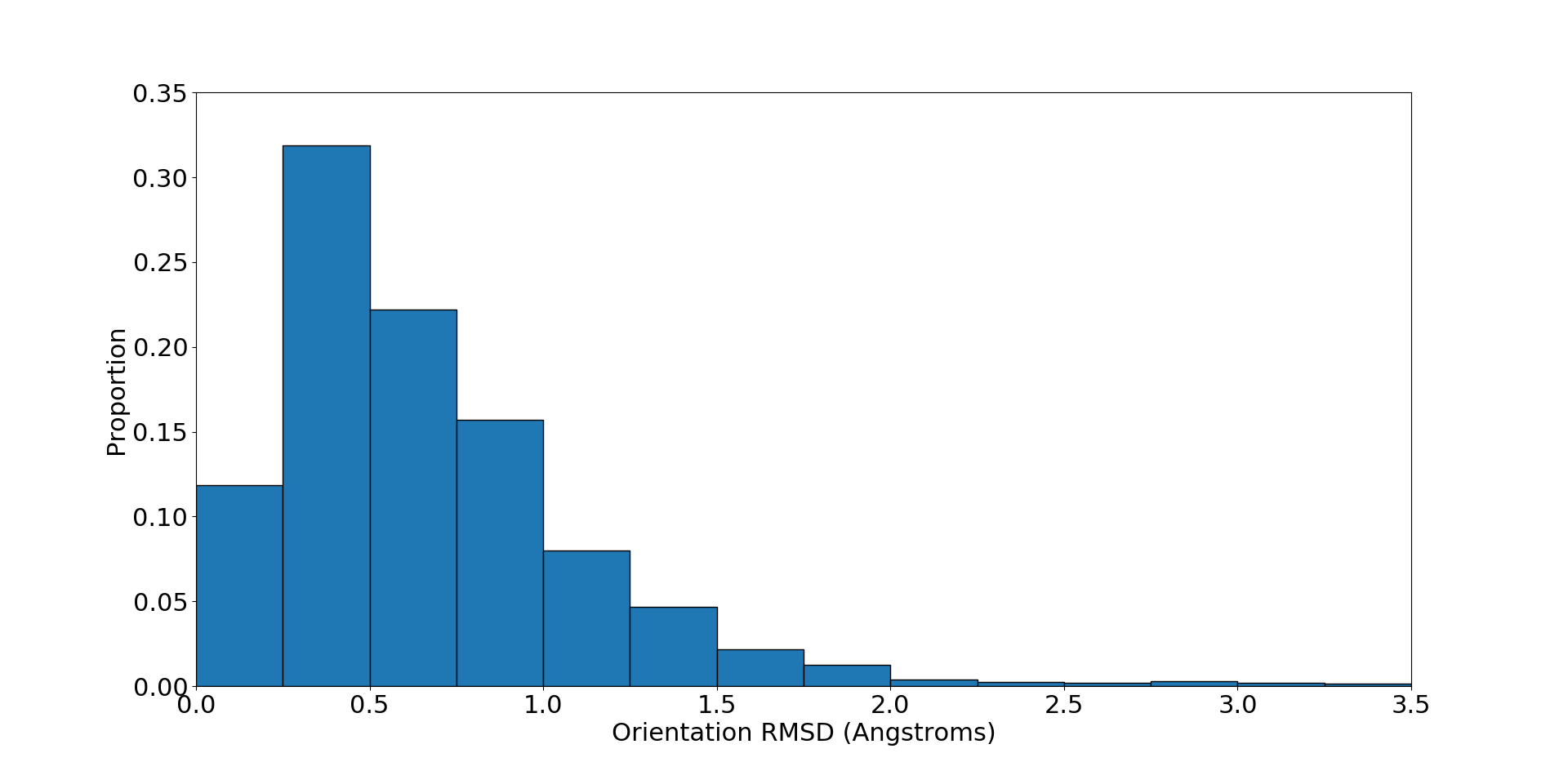

Supplement: S2 Fig — The distribution of orientation RMSDs observed between Fvs of identical heavy and light chain sequence. The vast majority (92%) have orientation RMSDs below 1.5Å (PNG) [file pcbi.1008781.s003.png]

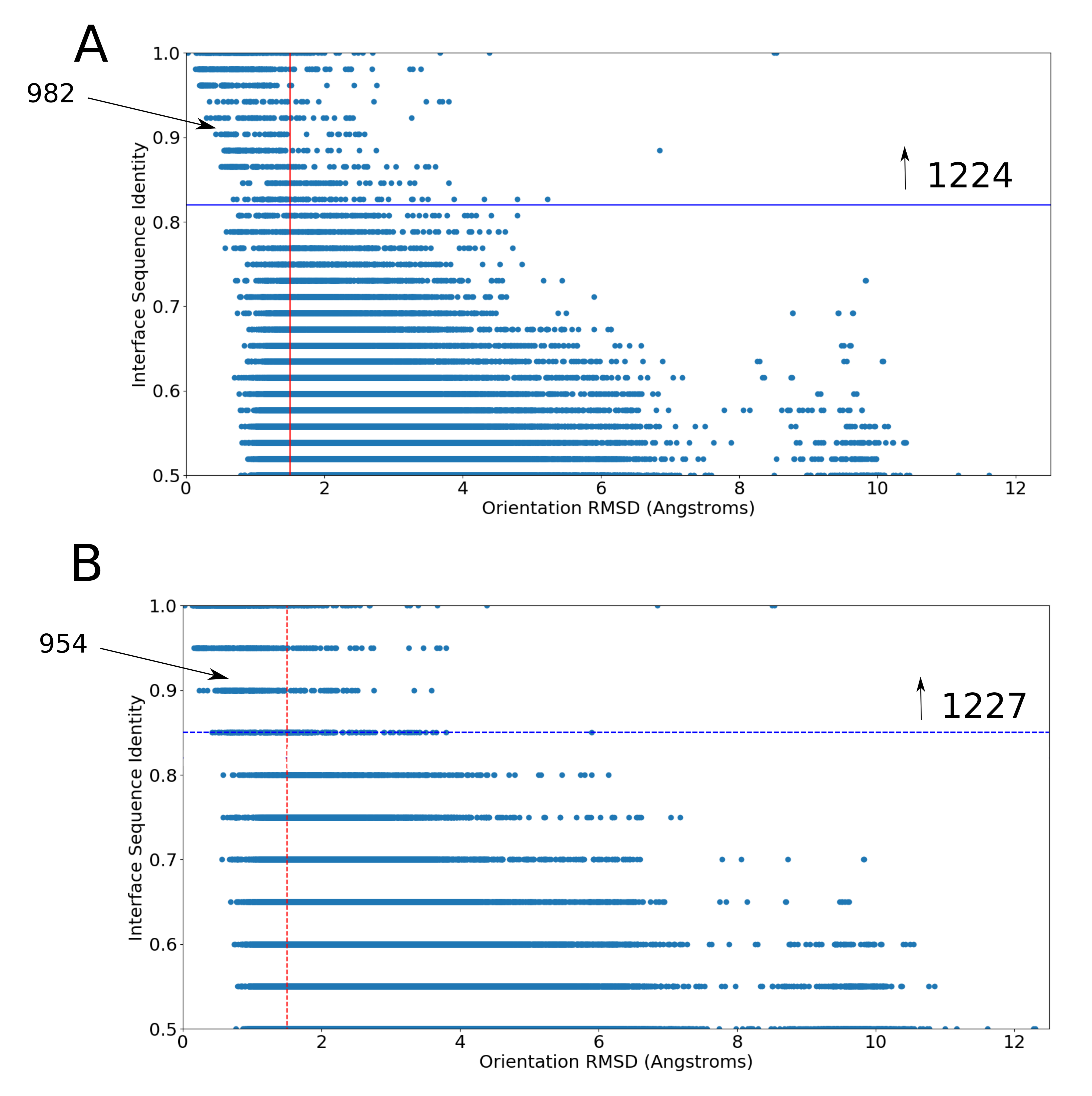

Supplement: S3 Fig — Graphs showing the orientation RMSD observed at each interface sequence identity value for (A) all 52 interface residues and (b) the 20 most important interface residues. The thresholds for (A) are set at 1.5Å and 82% sequence identity, while for (B) are set at 1.5Å and 85% sequence identity. The proportions above the sequence identity threshold and within 1.5Å orientation RMSD are 80.2% (982/1224) and 77.8% (954/1227) respectively. (PNG) [file pcbi.1008781.s004.png]

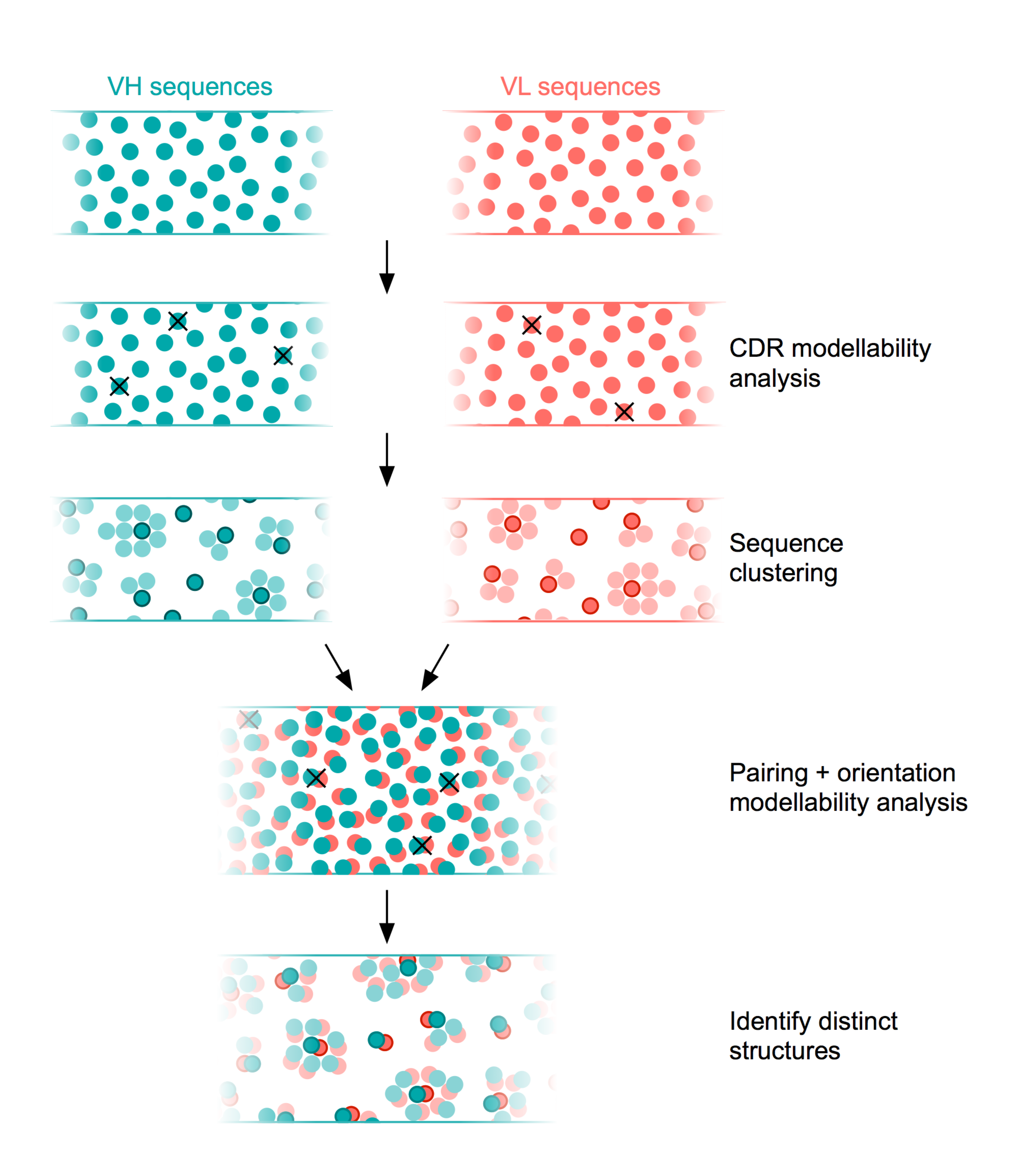

Supplement: S4 Fig — Heavy (VH) and light (VL) chain sequences from a repertoire snapshot are first analysed separately for their FREAD modellability (unmodellable chains are crossed out). They are then clustered by sequence identity using CD-HIT (90% threshold) for computational tractability. All VH and VL cluster centre chains are subsequently paired, and VH-VL orientations that cannot reliably modelled are removed (again shown by crosses). Finally, predicted modellable Fvs with identical combinations of CDR lengths are structurally clustered to identify ‘distinct structures’. (PNG) [file pcbi.1008781.s005.png]

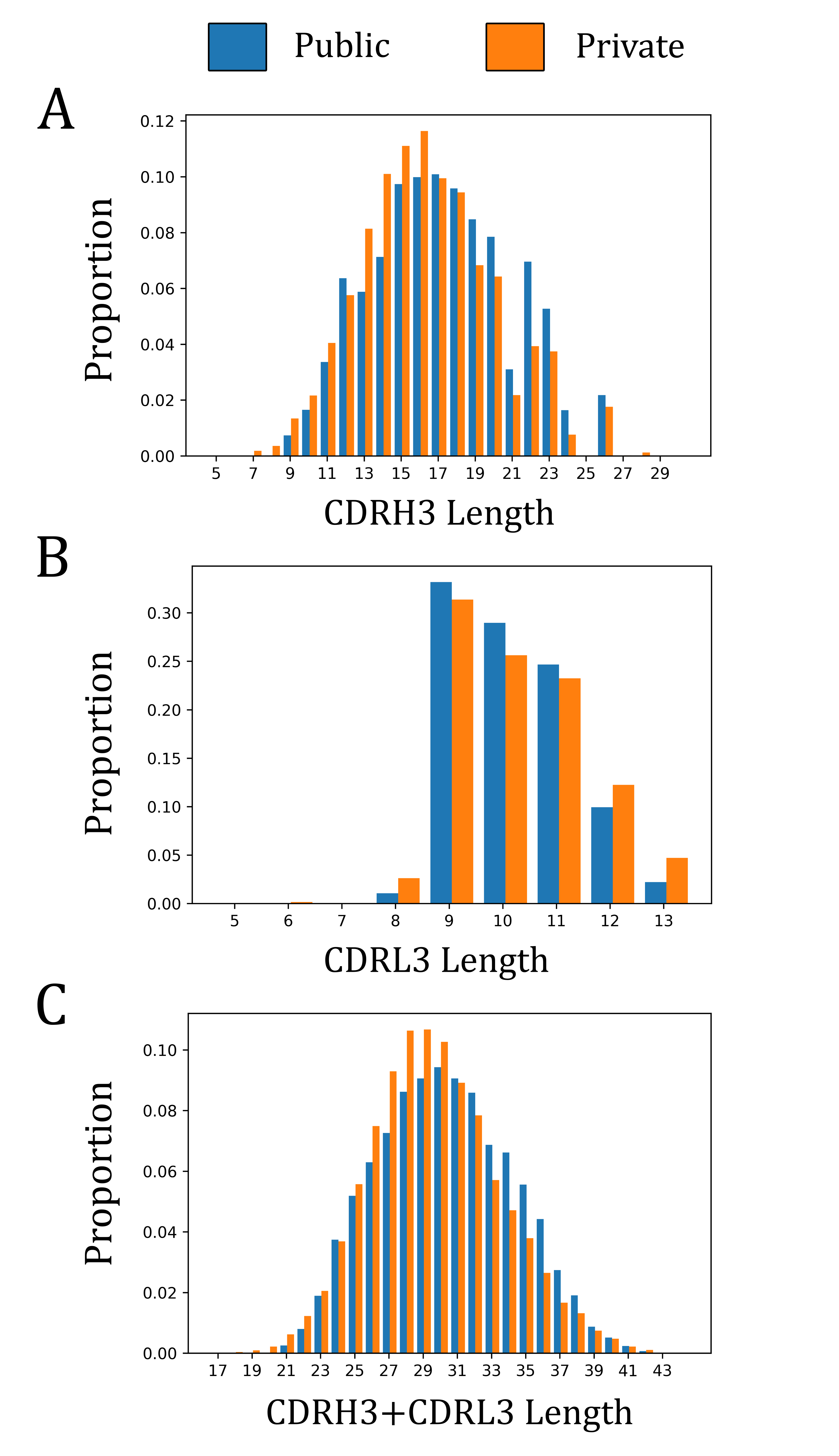

Supplement: S5 Fig — Bar charts comparing the (A) CDRH3 lengths, (B) CDRL3 lengths, (C) Combined CDRH3+CDRL3 lengths of S64 sequences assigned to ‘Public Baseline’ structures (blue) against those assigned to ‘Private Baseline’ structures (orange). (PNG) [file pcbi.1008781.s006.png]

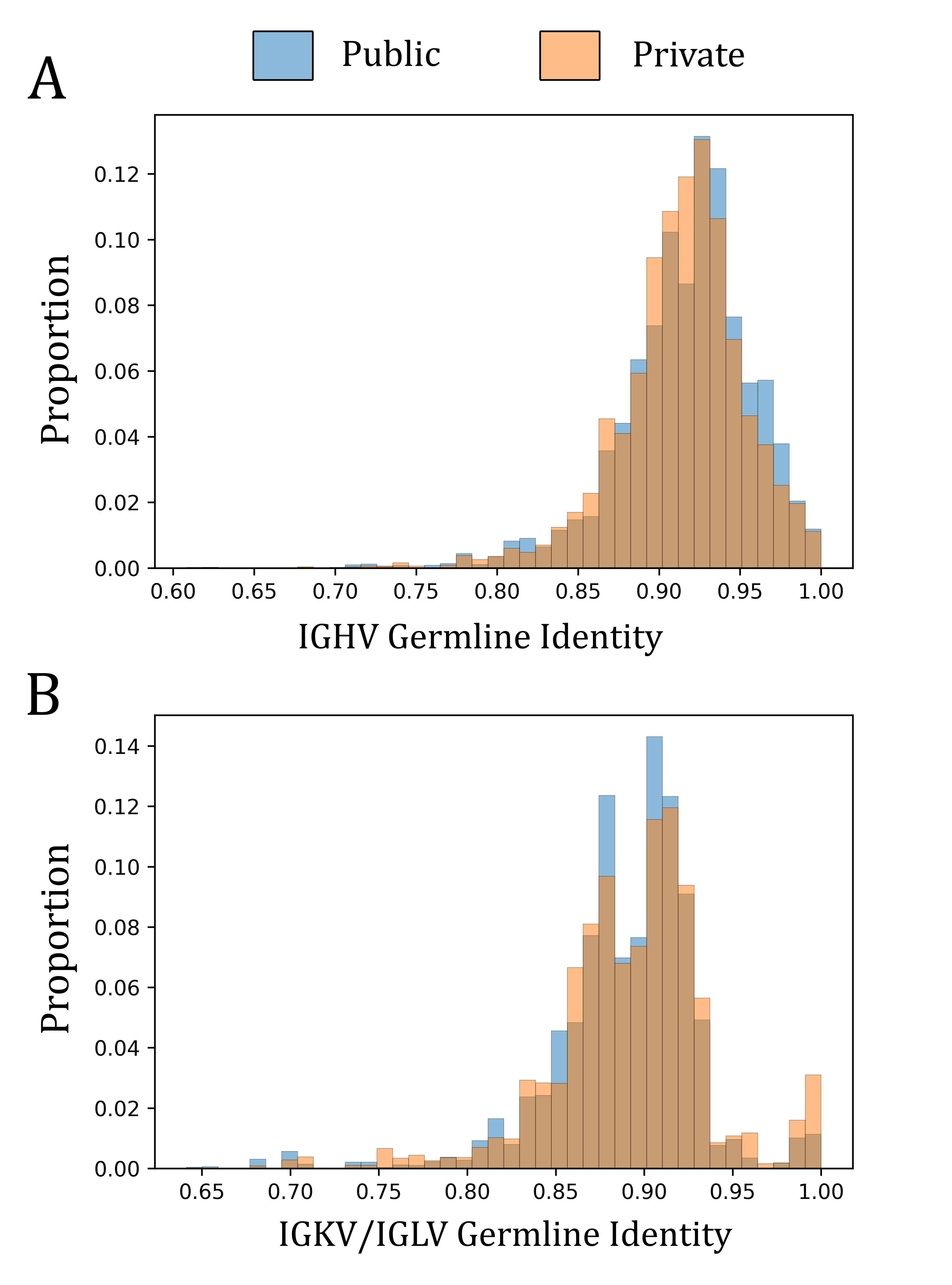

Supplement: S6 Fig — Histograms comparing the (A) closest IGHV germline sequence identity, and (B) closest IGKV/IGLV germline sequence identity of S64 sequences assigned to ‘Public Baseline’ structures (blue) against those assigned to ‘Private Baseline’ structures (orange). (PNG) [file pcbi.1008781.s007.png]

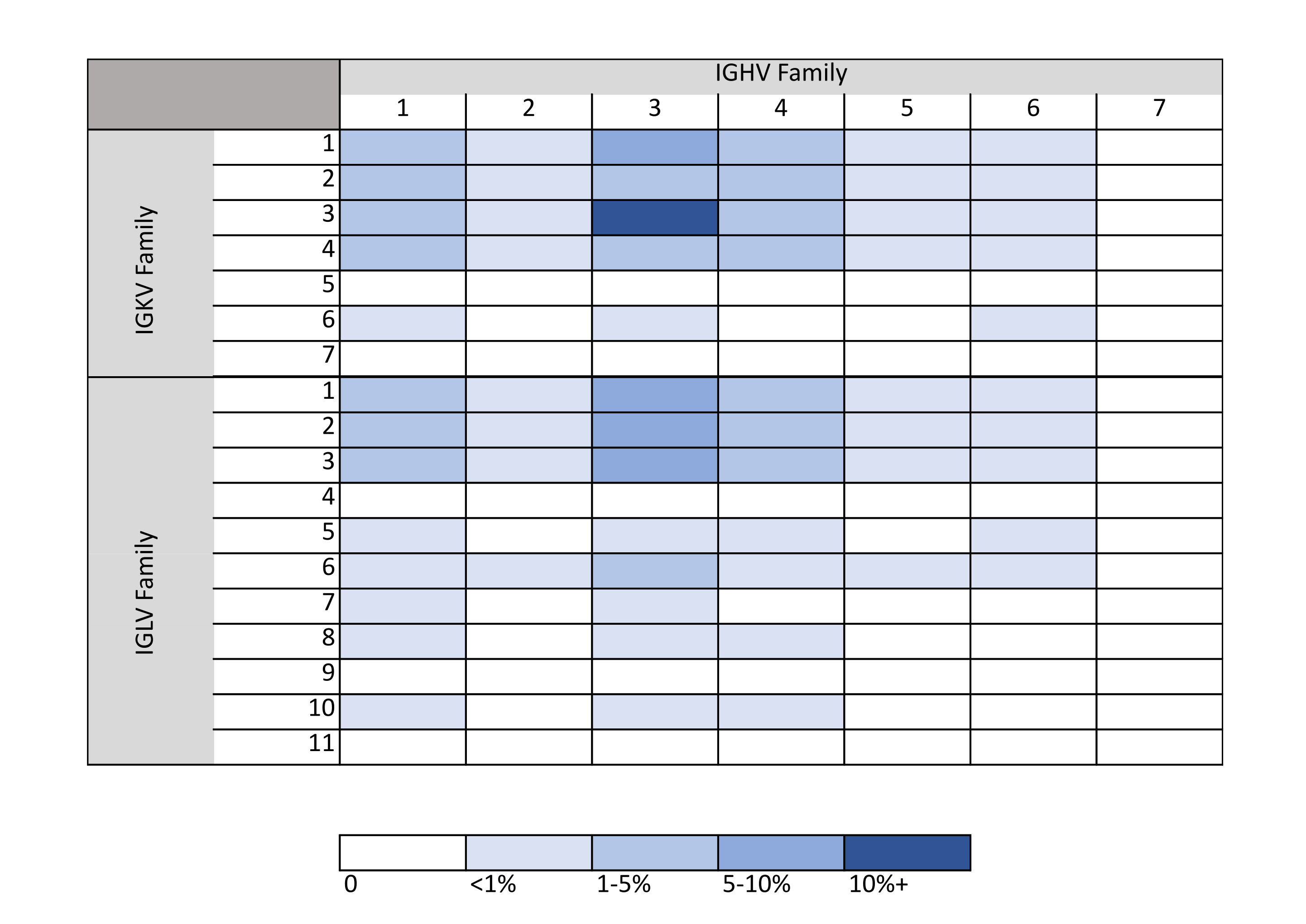

Supplement: S7 Fig — A heatmap showing IGHV:IGKV/IGLV gene family pairings across the ‘Public Baseline’ structures. The usage trends are consistent with the natural pairings observed in DeKosky et al. [11]. (PNG) [file pcbi.1008781.s008.png]

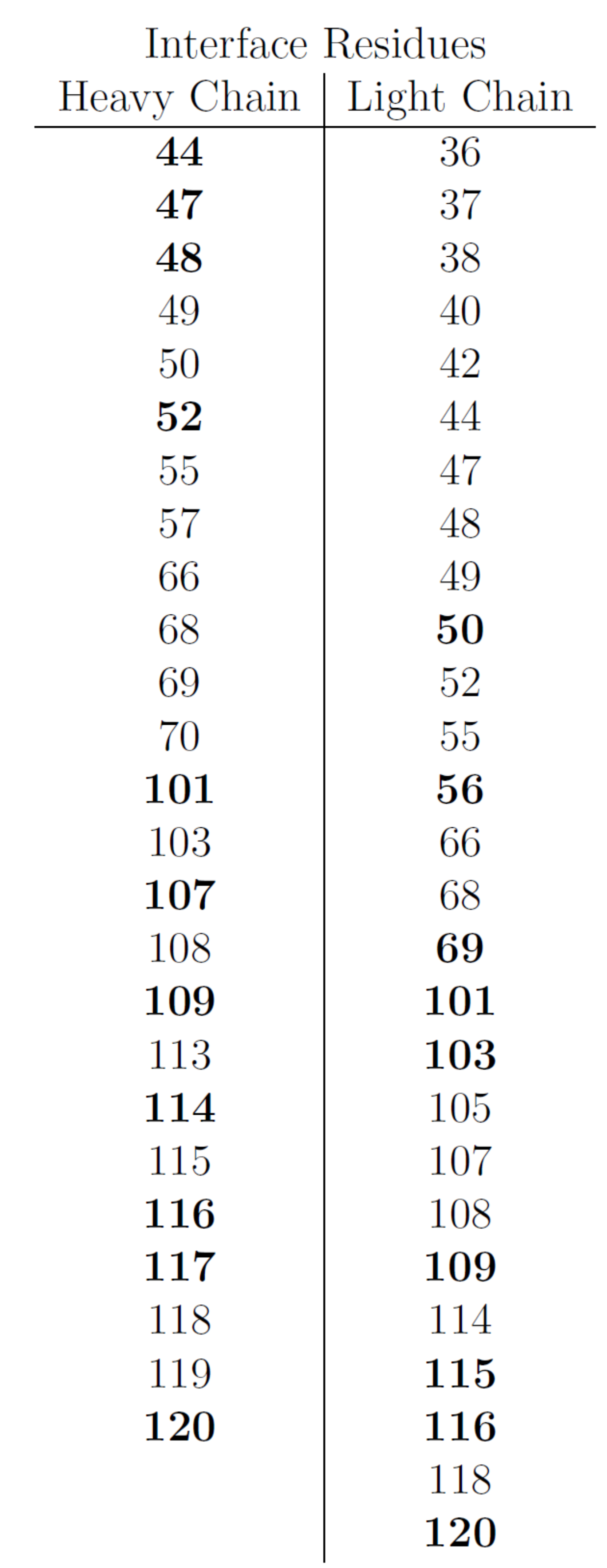

Supplement: S1 Table — The 52 heavy and light chain residues tending to lie in the heavy-light chain interface. Residue numbers in bold were determined to be amongst the five most important in the Random Forest regression model when predicting the six different ABangle parameters. (PNG) [file pcbi.1008781.s009.png]

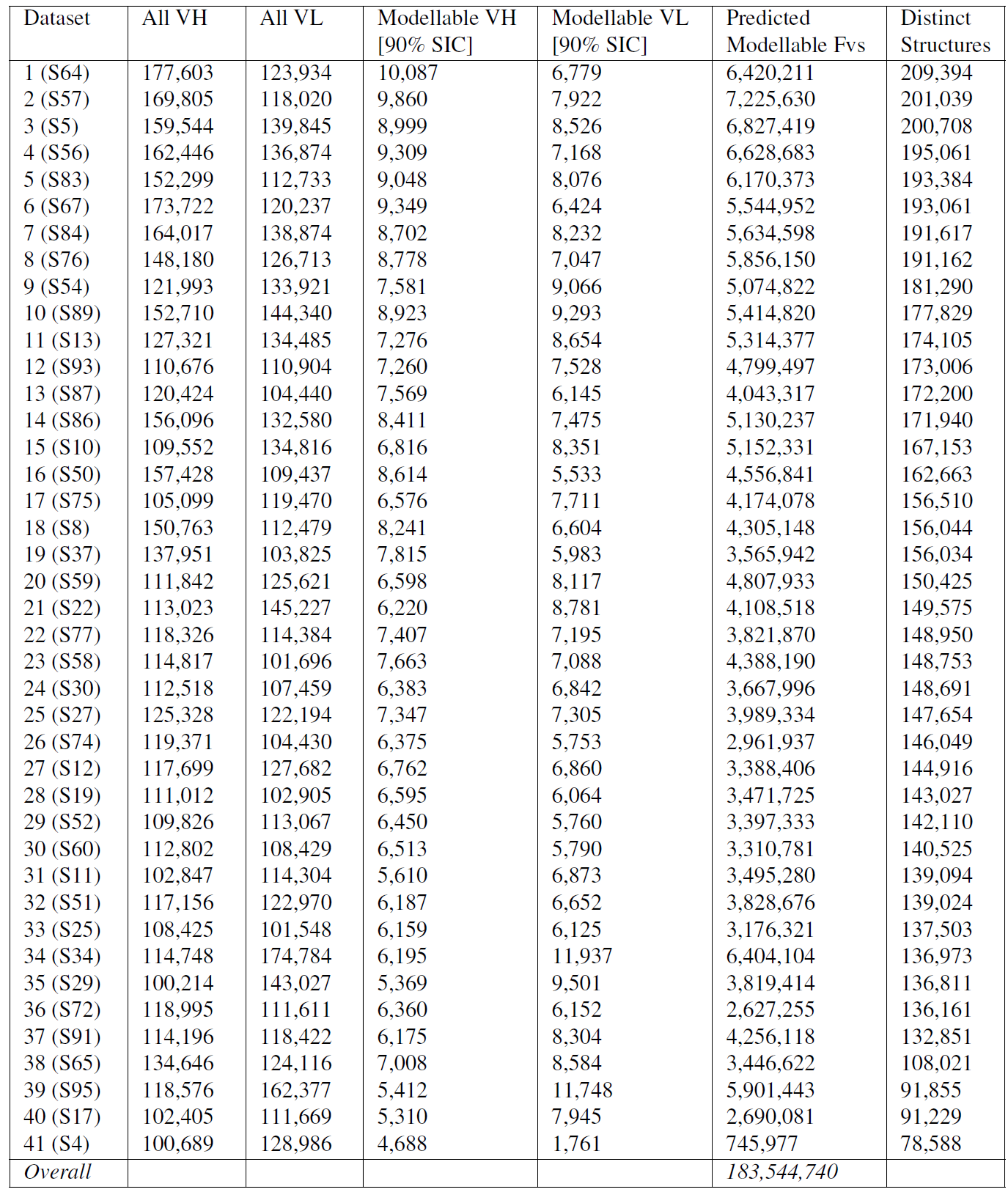

Supplement: S2 Table — Structurally profiling the baseline repertoire snapshots of 41 unrelated individuals. In order, the columns show: the dataset label, the number of VH and VL reads within each snapshot, the number of FREAD-modellable VH and VL reads (once clustered at 90% sequence identity), the number of predicted modellable Fvs resulting from these VH-VL pairings, and the number of distinct structures (cluster centres) identified in each dataset. SIC = Sequence Identity Clustered. (PNG) [file pcbi.1008781.s010.png]

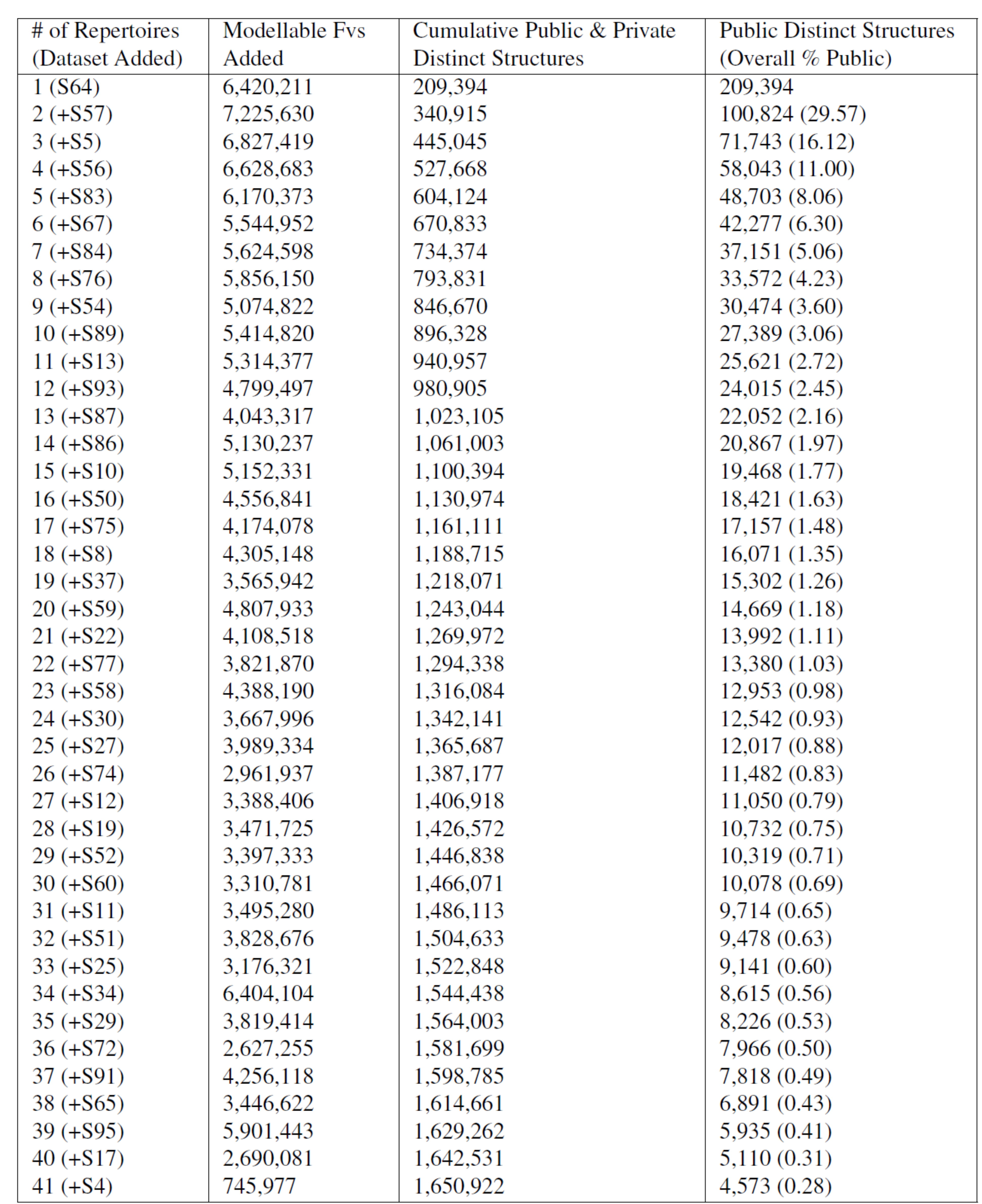

Supplement: S3 Table — Evaluating the number of public distinct structures seen across multiple baseline repertoire snapshots. In order, the columns show: the number of repertoires compared (in brackets the identifier of the last dataset added), the number of predicted modellable Fvs added by the last dataset, the number of distinct structures added by the last dataset, the (cumulative) number of public and private distinct structures across all compared repertoires, and the number of proportion of these structures that are public. The sharp drop-off in the proportion of public structures in the final four repertoire snapshots can be rationalised by their substantially lower internal structural diversity (see Table 2). (PNG) [file pcbi.1008781.s011.png]

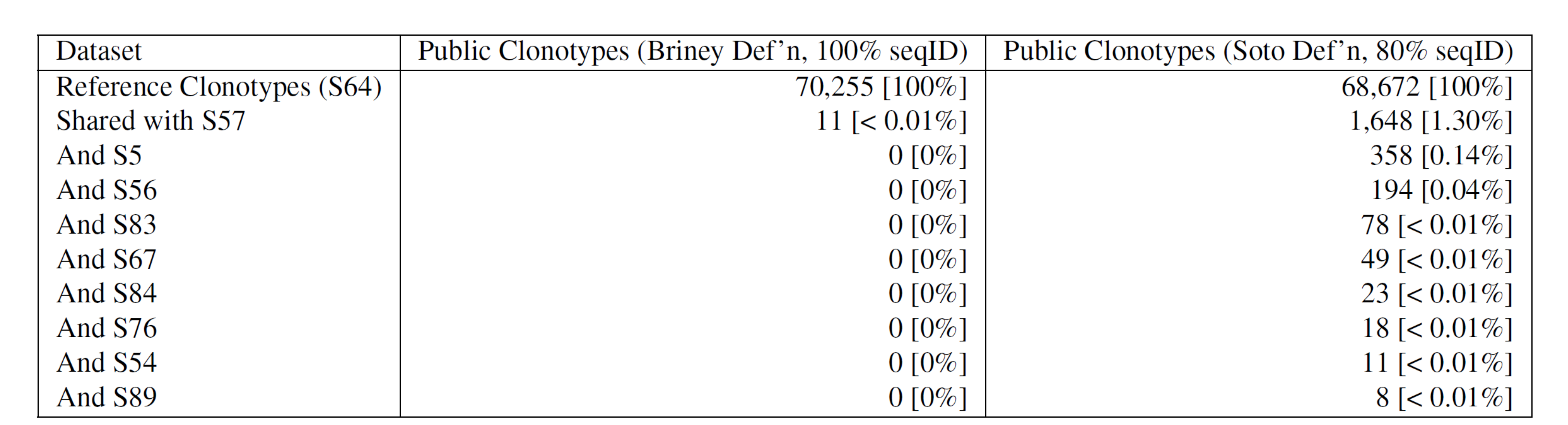

Supplement: S4 Table — Tracking the number of public clonotypes shared across all naïve baseline datasets analysed up to that point (e.g. 358 clonotypes are present in S64, S57, and S5 according to the Soto V3J definition). (PNG) [file pcbi.1008781.s012.png]

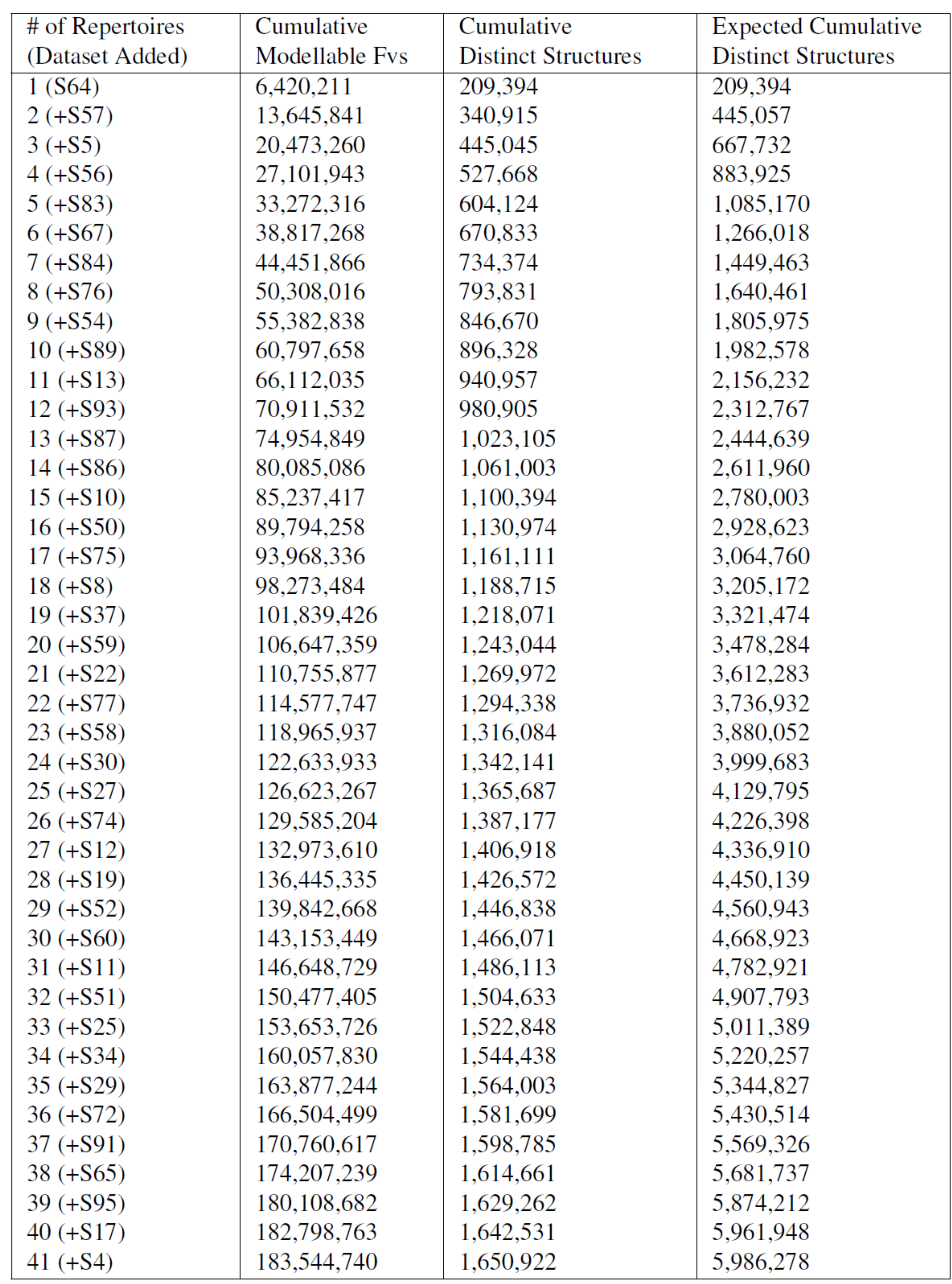

Supplement: S5 Table — Tracking the total number of public and private distinct structures seen across multiple baseline repertoire snapshots. In order, the columns show: the number of repertoires compared (in brackets the identifier of the last dataset added), the cumulative number of predicted modellable Fvs, the number of public and private distinct structures seen across all compared repertoires, and the expected number of cumulative public and private distinct structures if new distinct structures were observed at the same rate per modellable Fv as seen in S64. (PNG) [file pcbi.1008781.s013.png]

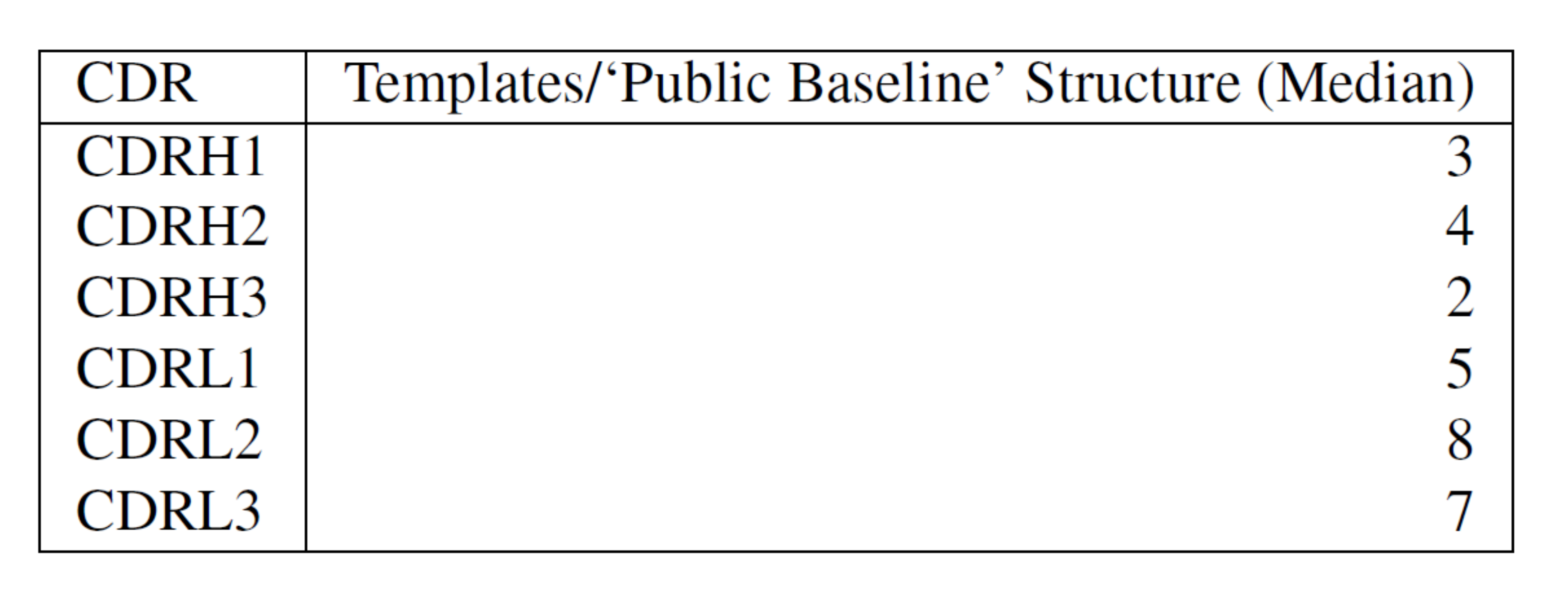

Supplement: S6 Table — The median numbers of unique FREAD templates assigned to each CDR within a ‘Public Baseline’ distinct structure. (PNG) [file pcbi.1008781.s014.png]

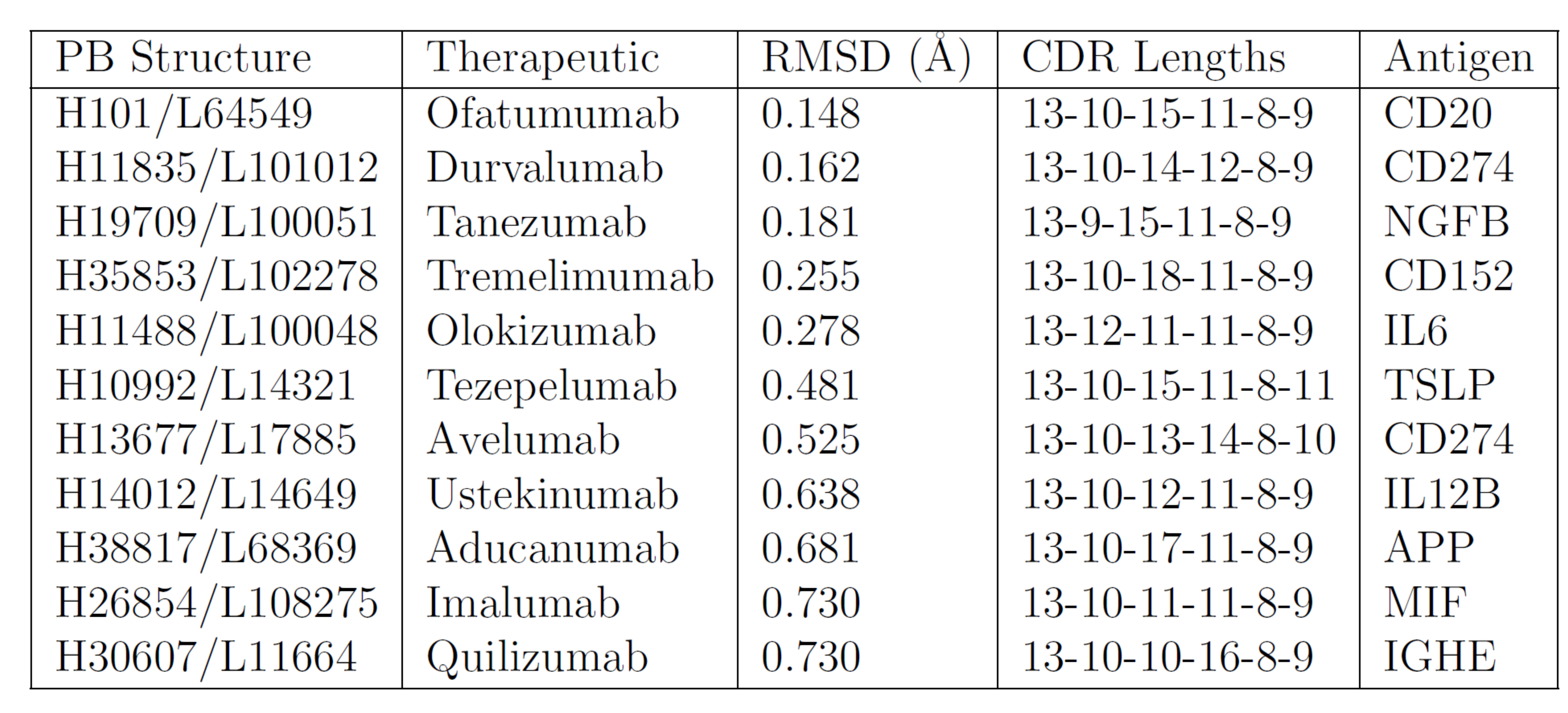

Supplement: S7 Table — The eleven clinical-stage therapeutic antibodies with a solved crystal structure within 0.75Å variable domain (Fv) root-mean-squared deviation (RMSD) of an antibody model structure from the Public Baseline Antibody Model Library (PB AML). The first column records the Fv identifier for the geometrically closest AML model to each of the eleven therapeutics listed in column 2. Column 3 provides the Protein Data Bank (PDB) identifier for each chosen therapeutic structure (chain identifiers in brackets). The corresponding RMSD is provided in column 4; all RMSD comparisons were made between AML structures and therapeutics with an identical combination of CDR lengths. This combination of North-defined CDR lengths is then listed in the order H1-H2-H3-L1-L2-L3. Finally, the target for each therapeutic antibody is recorded. PDB = Protein Data Bank; VH = variable heavy chain; VL = variable light chain; Fv = Fragment variable region; RMSD = root-mean-squared deviation; CDR = Complementarity-Determining Region. Antigens: CD—Cluster of Differentiation protein, NGFB—Nerve Growth Factor B, IL—interleukin, TSLP—Thymic Stromal Lymphopoietin, APP—Amyloid Precursor Protein, MIF—Macrophage Migration Inhibitory Factor, IGHE—Immunoglobulin Heavy Constant Epsilon. (PNG) [file pcbi.1008781.s015.png]

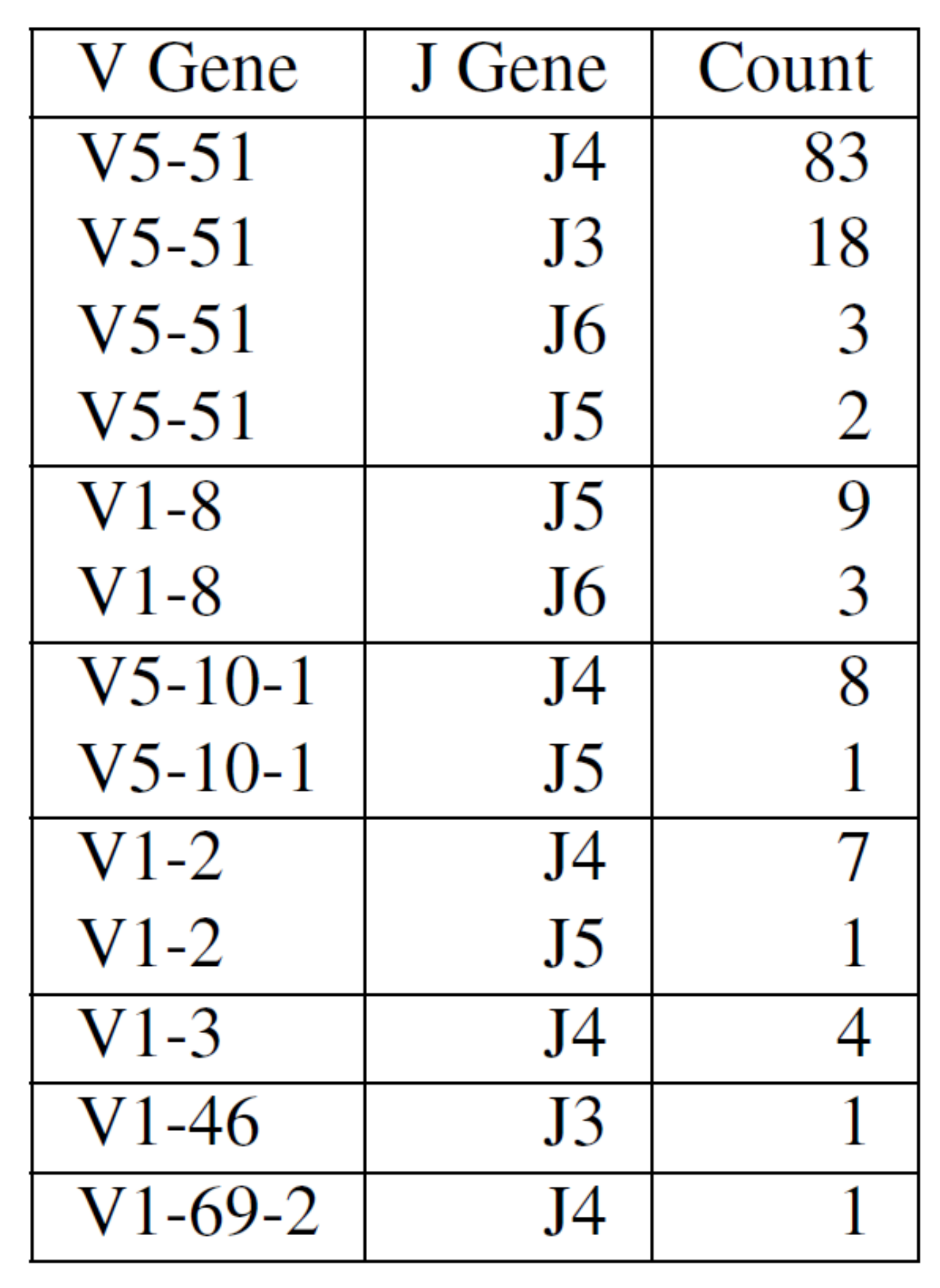

Supplement: S8 Table — The diversity of IGHV/IGHJ gene combinations represented across the 141 VH clonotypes assigned by Repertoire Structural Profiling to the ‘H14012+L14649’ ‘Public Baseline’ distinct structure. (PNG) [file pcbi.1008781.s016.png]

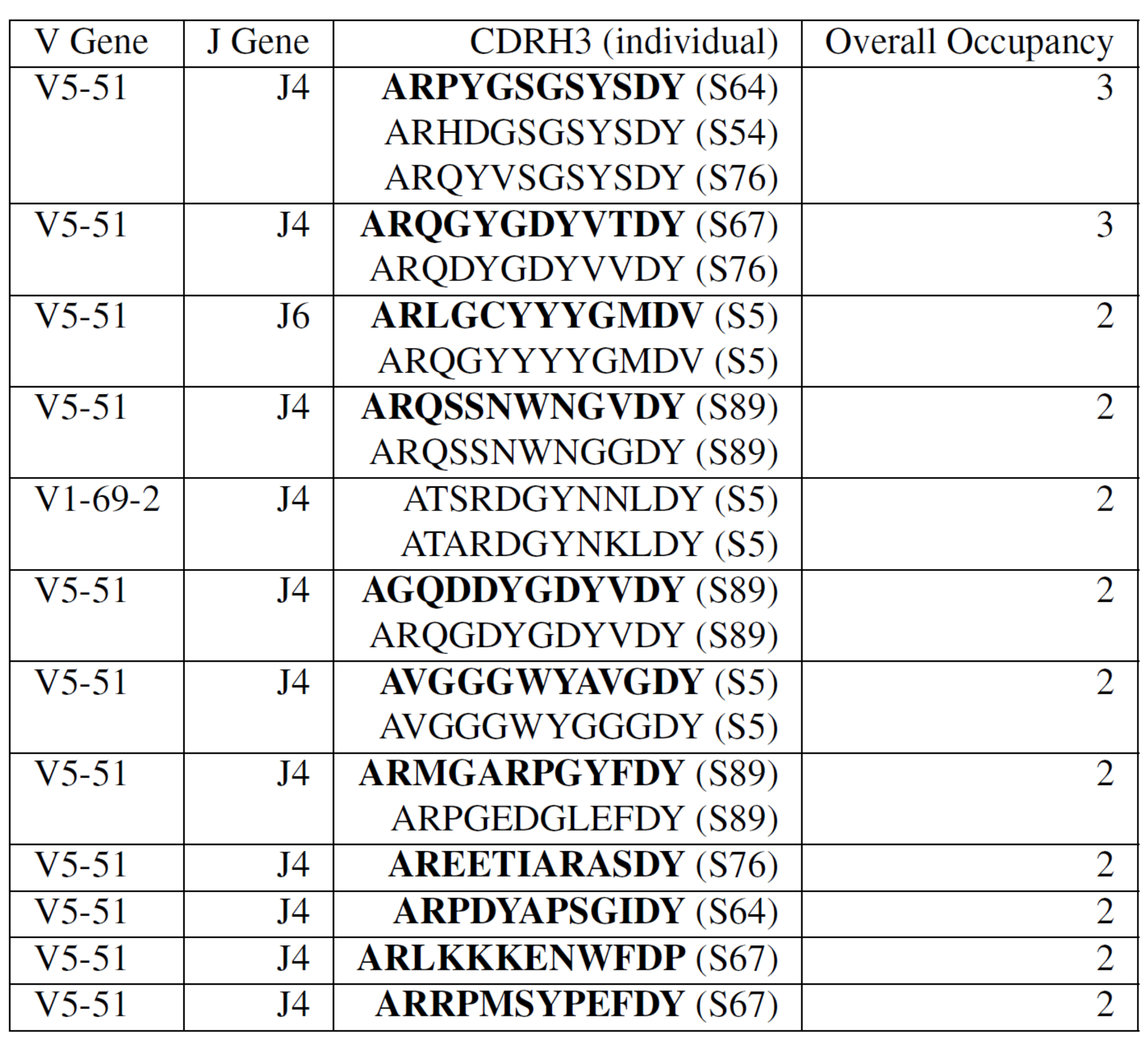

Supplement: S9 Table — The 12 multiple-occupancy VH clonotypes assigned by Repertoire Structural Profiling to the ‘H14012+L14649’ ‘Public Baseline’ distinct structure. (PNG) [file pcbi.1008781.s017.png]

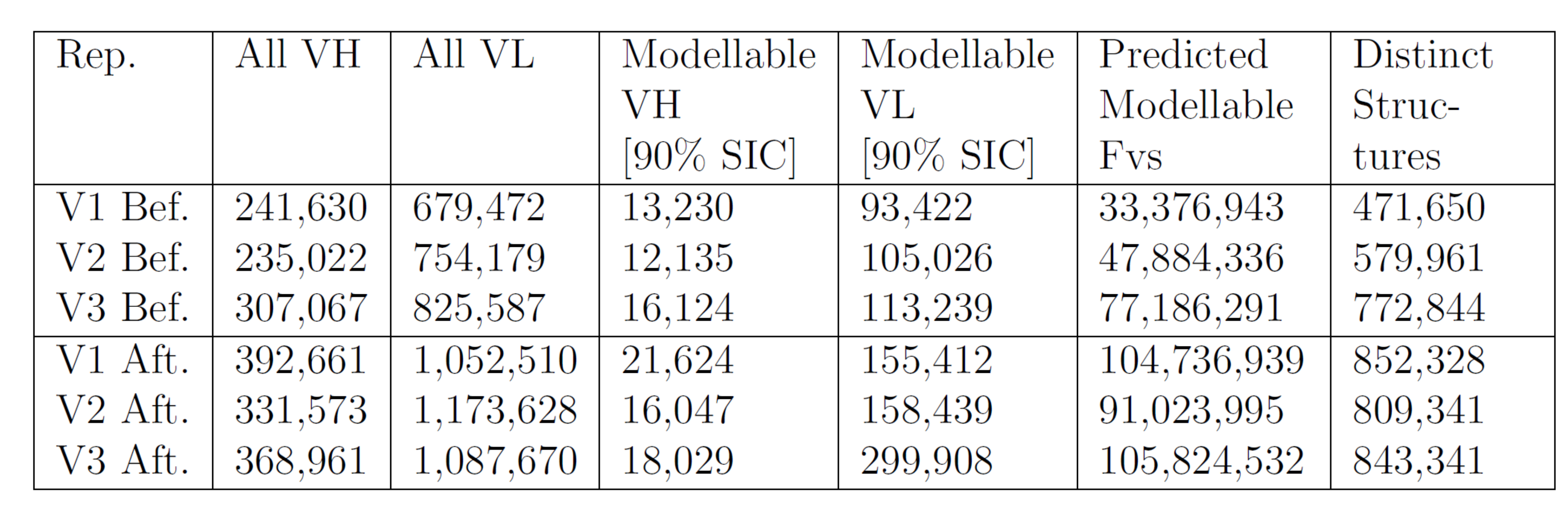

Supplement: S10 Table — Structurally profiling the ‘Before Vaccination’ (Before) and ‘After Vaccination’ (After) repertoire snapshots of three unrelated individuals (V1, V2, and V3). In order, the columns show: the dataset label, the number of VH and VL reads within each snapshot, the number of FREAD-modellable VH and VL reads (once clustered at 90% sequence identity), the number of predicted-modellable Fvs resulting from these VH-VL pairings, and the number of distinct structures (cluster centres) identified through greedy structural clustering. SIC = Sequence Identity Clustered. (PNG) [file pcbi.1008781.s018.png]

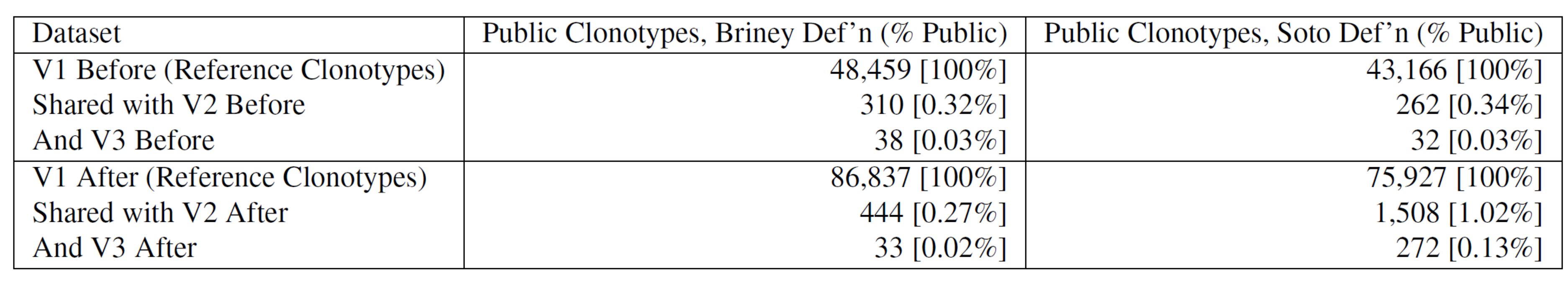

Supplement: S11 Table — Tracking the number of public clonotypes shared across all “Before Vaccination” (Before) datasets and all “After Vaccination” (After) analysed up to that point (e.g. 272 clonotypes are public across V1, V2, and V3 according to the Soto V3J definition). The Briney definition clusters CDRH3s at 100% sequence identity and same V/J genes, while the Soto Definition clusters CDRH3s at 80% sequence identity and same V/J genes. (PNG) [file pcbi.1008781.s019.png]
