# Supplementary material for: Public Baseline and shared response structures support the theory of antibody repertoire functional commonality
Source: PLoS Comput Biol. 2021 Mar 1;17(3):e1008781. doi: 10.1371/journal.pcbi.1008781 (PMC7951972; doi:10.1371/journal.pcbi.1008781)
Supplement: Attachment — Submitted filename: Reply_To_Reviewers_140920_FINAL.pdf [file pcbi.1008781.s020.pdf]

Dear Professor Dunbrack,

Editor's Comments

While the reviewers all appreciated the goals of the study and the significant efforts employed in the analysis presented in the paper, they share some misgivings on the interpretation of the results and whether the results could be an artifact of the necessary filters and cutoffs employed in defining "modellable" structures and the method for determining a random repertoire.

We thank the editor and reviewers for their thorough feedback on our manuscript, which we believe has significantly strengthened the work. We have responded fully to all comments raised in a point-by-point fashion below. All changes to the manuscript have been described and are marked in red in an attached PDF.

(**A**) I have similar concerns. In particular, I wonder if the random repertoire is affected by not filtering the templates for human structures of CDRH1/2, CDRL1/2, CDRH3, and CDRL3. I don't understand the utility of including non-human structures in generating the random repertoire.

We thank the editor for raising this interesting point. Through internal benchmarking we have established that including "non-human" templates in our FREAD loop databases leads to greater structural coverage and a net improvement in CDR structure prediction accuracy. This improved CDR loop structure prediction translates to better ABodyBuilder Fv models. We also note that:

1. Given the highly engineered nature of the PDB, and the fact that each PDB entry has just a single "organism" tag, organism origin labels are often misleading. For example, a chimeric structure that possesses mouse CDRs but a human framework region will only get one species origin label, and it is entirely up to individual researchers as to whether it is labelled as "mouse" or "human".

2. Previous work in our group to develop a sequence-based canonical form predictor (SCALOP, Wong *et al*., Bioinformatics, 2019) showed that the canonical backbone structures for H1-2, L1-3 are invariant to PDB organism label.

3. Previous work in our group has investigated the frequency with which the CDRH3 sequences in BCR-seq data are assigned to templates of different species (Kovaltsuk *et al*., PLOS Comp Bio, 2020). Their implementation of FREAD assigns "mouse" CDRH3 templates as the best template to model human IgM and IgG BCR-seq data (CDRH3 Lengths ≤ 16) roughly proportionately to their abundance in the FREAD CDRH3 loop database. This is shown in the left-hand-side of Figure S4 of their paper, where the dashed line symbolises the expected usage by species origin based solely on random sampling of the FREAD database.

The above demonstrates why excluding a template solely based on its PDB organism label would not be beneficial to the analysis.

However, there will be some true underlying species biases (e.g. natural mouse/camelid CDRH3 backbone conformations that are impossible to replicate incorporating human D genes). There may also be biases reflecting templates FREAD is willing to pick, primarily those with anchor residues that do graft convincingly onto any human framework template. Therefore, to account for both of these biases we rebuilt the Random Repertoires adding the extra criterion that only Fv and CDR templates that have previously been assigned to a human BCR-seq sequence in the Gidoni or Gupta data can be selected (Table R1).

The Random Repertoires now only have access to:

663 Fv templates
789 H1+H2 combinations of which around 55% are “human”, 30% are “mouse”, and 15% are “other”
2,051 H3 templates of which around 50% are “human”, 40% are “mouse”, and 10% are “other”
912 L1+L2 combinations of which around 67.5% are “human”, 25% are “mouse”, and 7.5% are “other”
2,125 L3 templates of which around 55% are “human”, 40% are “mouse”, and 5% are “other”

The corresponding template PDB-reported species distributions are listed in Table R2. This new restriction has the effect of upweighting the proportionate use of “human origin” templates from their abundances in the raw FREAD database, suggesting a degree of correction for true species conformation bias. We note that the proportion of “mouse origin” templates remains significant – we consider this reflects both that a proportion of binding site geometries are accessible to both species and that PDB species assignments have low reliability.

CDR	Total Number of FREAD Templates	Proportion of FREAD templates labelled as “human”	Number of FREAD Templates Assigned to a Human BCR sequence	Of which derive from the same PDB structure (V gene restriction)
H1	2,526	37.99%	1,079 (42.7%)	789
H2	2,575	37.85%	1,615 (62.7%)	
H3	2,502	36.47%	2,051 (82.0%)	n/a
L1	2,355	46.85%	1,172 (49.8%)	912
L2	2,373	47.00%	1,546 (65.1%)	
L3	2,376	47.07%	2,125 (89.4%)	n/a

Table R1. The proportion of each CDR FREAD database assigned to a human Gidoni or Gupta BCR-seq sequence.

CDR	Proportion of FREAD templates labelled as “human”	FREAD templates assigned to a human BCR sequence	“Human” Origin	“Mouse” Origin	“Other Origin”
H1	37.99%	789	461 (58.4%)	213 (27.0%)	115 (14.6%)
H2	37.85%				
H3	36.47%	2051	1006 (49.0%)	814 (39.7%)	231 (11.3%)
L1	46.85%	912	618 (67.7%)	225 (24.7%)	69 (7.6%)
L2	47.00%				
L3	47.07%	2125	1204 (56.7%)	814 (38.3%)	107 (5.0%)

Table R2. The species origin biases across the new Random Repertoire template choices.

We recalculated all values related to RRs in the manuscript adding this new restriction. The number of distinct structures in each Fv-sample size matched RR reduced by an average of 90,000 (Table R3) however this still represents a much greater structural diversity than each corresponding real repertoire. When translated to expected overlaps, each new Random Repertoires overlaps more than the previous ones, although still at a much lower level than real repertoires (Table R4).

Previously, we erroneously expressed the “Expected % Public Distinct Structures” using the denominator of “cumulative number of distinct structures across N **real** repertoires. This has now

been fixed to use the denominator of “cumulative number of distinct structures across N **random** repertoire subsamples. This pushes the expected percentage public structures to a slightly lower value, but the same trend (sharp exponential decrease) is observed.

--

The 183M “Modellable Repertoire Structures” clustered into *c.* 24.4M distinct structures over *c.*39.9K distinct CDR length combinations (compares with 28.7M distinct structures over 61.1K distinct CDR length combinations before).

When restricted to just the 3,468 S64 CDR length combinations, *c.* 154.4M Modellable Repertoire Structures remained, which clustered into *c.* 18.0M distinct structures (compares with 154.8M clustering to *c.* 20.8M distinct structures before)

Individual	Number of Samples	New Random Repertoire Distinct Structures (Former values scored)	Corresponding Real Repertoire Distinct Structures
S64	6,420,211	2,092,117 (~~2,187,257~~)	209,394
S57	7,225,630	2,256,899 (~~2,365,651~~)	201,039
S5	6,827,419	2,175,112 (~~2,267,554~~)	200,708
S56	6,628,683	2,114,341 (~~2,216,066~~)	195,061
S83	6,170,373	1,994,931(~~2,089,996~~)	193,384
S67	5,544,952	1,868,469 (~~1,949,297~~)	193,061
S84	5,624,598	1,907,994 (~~1,997,314~~)	191,617
S76	5,856,150	1,941,503 (~~2,024,832~~)	191,162
S54	5,074,822	1,710,035 (~~1,781,630~~)	181,290
S89	5,414,820	1,813,336 (~~1,892,597~~)	177,829

Table R3. The new numbers of distinct structures observed over Random Repertoires, given the new Fv/CDR template restrictions. As expected, the structural diversity drops.

# of Repertoires (Dataset Added)	Cumulative Public & Private Distinct Structures	Public Distinct Structures (Overall % Public)	Expected Public Distinct Structures, Ovr % Public (Previous Exp. # scored out)
1 (S64)	209,394	209,394	209,394
2 (+S57)	340,135	100,824 (29.57%)	12307, 3.10% (~~10455~~)
3 (+S5)	445,045	71,743 (16.12%)	1600, 0.28% (~~1351~~)
4 (+S56)	527,668	58,043 (11.00%)	322, 0.04% (~~293~~)
5 (+S83)	604,124	48,703 (8.06%)	86, <0.01% (~~78~~)
6 (+S67)	670,833	42,277 (6.30%)	31, <0.01% (~~32~~)
7 (+S84)	734,374	37,151 (5.06%)	17, <0.01% (~~16~~)
8 (+S76)	793,831	33,572 (4.23%)	9, <0.01% (~~9~~)
9 (+S54)	846,670	30,474 (3.60%)	6, <0.01% (~~5~~)
10 (+S89)	896,328	27,389 (3.06%)	4, <0.01% (~~4~~)

Table R4. The new expected numbers of shared distinct structures based on sampling the new Random Repertoires. As expected, the degree of expected sharing increases.

The following manuscript changes have been made:

> **METHODS**: The description of building Random Repertoires has changed to include the new restrictions. The numbers of available Fv/CDR templates have been changed to reflect the new restrictions.
CDR Modellability Analysis
Templates were not restricted only to those with "human" PDB organism assignments for multiple reasons. Antibodies in the PDB are highly engineered, both through point residue mutations and entire loop transplantation, meaning organism origin labels are only accurate for a small number of entries. In addition, internal benchmarking of FREAD (Choi et al 2010, Choi et al 2011) and ABodyBuilder (Leem et al 2016) showed that including "non-human" templates in our FREAD loop databases (particularly the CDRH3 database) leads to greater structural coverage and a net improvement in CDR structure prediction accuracy.
> **RESULTS**: All statistics in the manuscript text related to Random Repertoires have changed to reflect the new datasets. Figures in the final column of Table 2 have been updated. Analysis and conclusions remain unchanged.
> Due to the inaccuracy of PDB species origin labelling and FREAD template biases, we have reconsidered and believe it is too strong to draw any conclusions from the fact that the human public baseline AML used a high proportion of "human-origin" templates. We have therefore removed this section of the analysis and replaced it with the more rigorous characterisation described below.

(**B**) I also wonder if the 3% Public Antibodies are closer to germlines than the private sequences, and may be overrepresented in the PDB. Some characterization of the properties of the 3% Public Antibodies may help to clarify this issue.

We thank the editor for this suggestion – we also believe that the paper would benefit from more extensive characterisation of the 3% public structure antibodies. In response, we have evaluated the CDR length distribution (Figure R1) and amino acid germline sequence identity (Figure R2) for the *c*. 27.3K "S64" Fv sequences assigned to public structures against the *c*. 182k "S64" Fv sequences assigned to private structures, both of which we have added to the Supporting Information.

Structures with long (L>20) CDRH3 loops form a higher proportion of the public baseline structures than they do of the private baseline structures, implying modellability bias may be artificially boosting the chances of a long-CDRH3 structure being assigned as public. As the structural space available to long CDRH3 (20+) loops is enormous, we have nowhere near full structural coverage with the small number of template structures available. Therefore if an Fv containing a long CDRH3 loop is considered modellable, it is more likely to be assigned to a structural template further away from its true structure, thus artificially overinflating the numbers of long CDRH3s that look structurally similar. We have added comment on this to our manuscript (see below changes to manuscript: *RESULTS>Characterising the Public Baseline Structures>CDR3 Length Usages*). Interestingly, Fv sequences adopting public baseline structures had very similar germline sequence identities to those adopting private distinct structures, suggesting that this is not a factor biasing modellability.

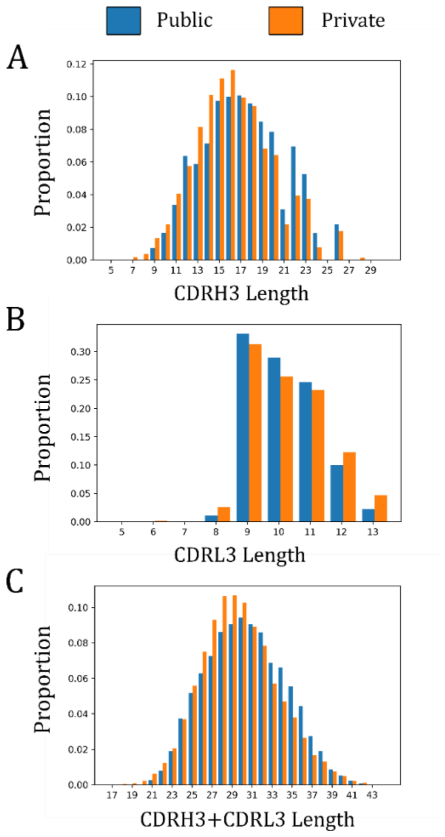

Fig. R1. A bar charts of (A) CDRH3 lengths, (B) CDRL3 lengths, and (C) CDRH3+CDRL3 lengths comparing the S64 sequences assigned to a public baseline structure (blue) to those assigned to a private structure (orange)

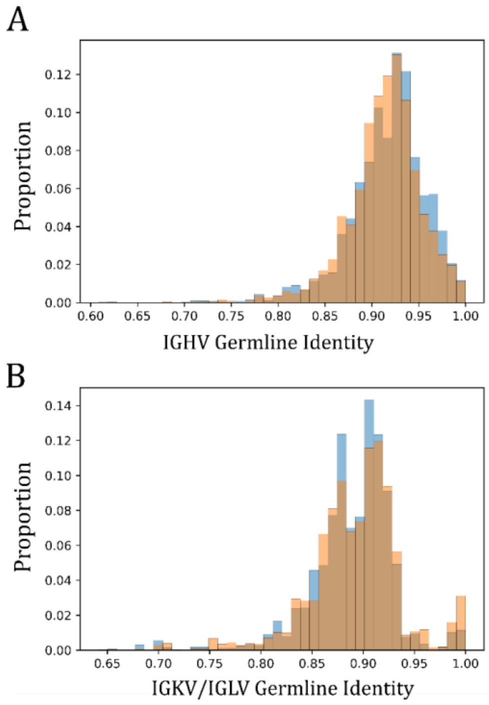

Fig. R2. Histograms comparing the sequence identity to germline across the S64 sequences assigned to Public (blue) vs. Private (orange) Baseline structures. (A) Sequence identity to the closest IGHV gene. (B) Sequence identity to the closest IGKV/IGLV gene.

For additional characterisation studies, we draw attention to our responses to the reviewers. In reply to Reviewer 2, we have shown that the IGHV:IG[K/L]V pairing frequencies used by the Public Baseline Repertoire are consistent with natural pairing frequencies seen in DeKosky *et al*. In the same reply, we have also shown an example of a Public Baseline structure and characterised the Fv sequences we assigned to it across the ten individuals. As part of this analysis, we performed lenient clonotyping on the Fv sequences to show that our method can pull together many antibodies that are unclusterable within a clonotyping framework (see Reviewer 2, Major Concern 3). In reply to Reviewer 3 we included a new dataset detailing the different FREAD CDR templates used in each distinct structure, to allow the structural spread within distinct structures to be investigated. In reply to Reviewer 4, we have evaluated the CDRL3 and CDRH3+CDRL3 length distributions of the `Public Baseline' vs. `Private Baseline' distinct structures, showing that no short H3-short L3 bias is evident in the `Public Baseline' set.

The following manuscript changes were made:

>RESULTS
Characterising the `Public Baseline' Structures
CDR3 Length Usages
"We compared the CDRH3, CDRL3 and CDRH3+CDRL3 distributions of the S64 Fv sequences assigned to a `Public Baseline' structure against those assigned to a `Private Baseline' structure. The CDRL3 and CDRH3+CDRL3 length usages demonstrate that `Public Baseline' structures are not an artefact of using shorter CDR3 loops with more limited conformations. In fact, we find that modellability bias is likely to be overstating the proportion of `Public Baseline' distinct structures with longer CDRH3 loop lengths (**SI Fig. 5**). The structural space available to long CDRH3 (20+) loops is enormous, and we have relatively poor structural coverage. As a result, if an Fv containing a long CDRH3 loop is considered modellable, it is more likely to be assigned to a structural template further away from its true structure, thus artificially inflating the numbers of long CDRH3s that look structurally similar. These longer CDR length `Public Baseline' structures should therefore be treated with caution and, as more templates of longer CDRH3 loops emerge improving CDRH3 modellability, we would expect their numbers to decrease to the public:private ratios seen at more moderate CDRH3 lengths."
Germline Proximity and Usages
"We also investigated whether S64 Fv sequences assigned to `Public Baseline' distinct structures were more proximal to germline than those assigned to `Private Baseline' structures (**SI Fig. 6**, see Methods). The germline proximity of both `Public' and `Private' Fvs to their closest IGHV and IG[K/L]V genes is very similar, indicating that `Public Baseline' structures are not solely an artefact of human V gene biases.
Finally, we considered the constituent paired V genes across the `Public Baseline' structures. As our pairing algorithm only predicts modellable Fv pairings based on PDB structures, we compared our IGHV/IG[K/L]V pairing frequencies with those observed in DeKosky *et al*.'s study of *c*. 2000 natively-paired antibodies (**SI Fig. 7**). Our `Public Baseline' gene pairing frequencies were very similar to DeKosky *et al*.'s native sample, with the IGHV1/IGKV1-4, IGHV1/IGLV1-3, IGHV3/IGKV1, IGHV3/IGKV3, and IGHV3/IGLV1-4 pairings the most abundant."
CDR Template Usages
We investigated the number of different structural templates that were assigned to each CDR in a `Public Baseline' distinct structure (SI Table 6). As expected, the lowest median number of different templates per distinct structure was recorded for the CDRH3 loop (2 templates/structure), consistent with the large structural variation within the region driving the definition of distinct binding site structures. Collectively, the light chain CDRs recorded considerably more FREAD

templates per structure (median of 20 templates/structure) than the heavy chain CDRs (median of 9 templates/structure). We have supplied the sets of FREAD templates assigned to each CDR of each distinct structure to facilitate further structural characterisations of distinct structures of interest (Dataset 1).

>RESULTS

Building and Characterising a `Public Baseline' Antibody Model Library

Proximity to Therapeutics

"To show that similar binding site residue profiles can also be found by Repertoire Structural Profiling, we examined `Public Baseline' distinct structure `H14012+L14649' as a case study (Fig. 3).

This structure lies within 0.64A of the therapeutic Ustekinumab (SI Table 5). Examining the backbone-aligned structures shows this difference lies in slightly different CDR loop structures assigned to the CDRH2, CDRH3, and CDRL3 loops (Fig. 3A). We then examined all 4,911 Fv sequences assigned to this distinct structure across the ten individuals (S64 through S89), looking for the closest CDR sequence identity matches to Ustekinumab. The most similar of the 155 sequence-unique VH sequences assigned to this distinct structure is shown in Fig. 3B. While both the Ustekinumab and `Public Baseline' VH sequences most closely aligned to the same V and J genes (IGHV5-51/IGHJ4), the CDRH3 sequences are only 66% sequence identical, and so would not have been assigned to the same VH clonotype (the typical minimum threshold is 80% as used in Soto *et al*. (Soto *et al*. 2019). This VH was coupled both with the VL sequence shown in Fig. 3B and with a VL sequence shown in Fig. 3C. The VL in Fig. 3B VL is more identical across the three CDRs (22/26, 85%), while the one in Figure 3C is closer in CDRL3 identity but considerably less so in CDRL2 identity. Both these VLs derive from different IGKV germlines to the Ustekinumab VL (Ustekinumab: IGKV1D-16, Fig. 3B VL: IGHV1-9, Fig. 3C VL: IGKV3-15). Overall, the Fv described in Fig. 3B is 75% sequence identical to Ustekinumab across all 6 CDRs.

This level of sequence and structural similarity between clinical-stage therapeutic antibodies and representatives of the `Public Baseline' repertoire suggests that Repertoire Structural Profiling could prove an effective tool for designing general screening libraries containing promising drug leads."

>DISCUSSION

"This might suggest that a large portion of structural commonality will remain across the (as yet) unmodellable regions of structural space, <u>although we do expect the number of `Public Baseline' structures with long CDRH3 loops to fall, as modellability may be increasing this figure</u>."

>Figure R3 (Ustekinumab *vs*. Public Baseline structure case study) has been added to the main manuscript as Figure 3.
>Figure R1 (comparison of CDR3 lengths) has been added as SI Fig. 5.
>Figure R2 (germline proximity histograms) has been added as SI Fig. 6.
>Figure R4 (comparison of heavy:light germline pairings to DeKosky) has been added as SI Fig. 7

(**C**) Finally, because the study is performed on non-paired VH and VL sequences, it is difficult to see how it is possible to demonstrate that vaccination results in antibodies that bind to the same epitopes in the vaccine in the same way (i.e, roughly they would have very similar antigen/antibody co-complex structures such that the paratope/epitope interface and orientation are similar). Are there any paired VH/VL antibody sequence sets that could be added to the analysis?

We thank the editor for this suggestion. Starting from unpaired data, it is impossible to conclusively prove a common variable domain (Fv) structure was raised in several individuals. However, we believe that we have shown it to be statistically extremely likely given the binding site combinations

that can be made before *vs*. after vaccination. Repertoire Structural Profiling captures the broadest possible structural diversity that can be generated from a set of modellable VH and VL chains in an unpaired repertoire. The way we constructed our longitudinal vaccination analysis was to look for structures that could not be generated from the component VH/VL sequences in any individual before vaccination, but that could be reconstructed in all individuals after vaccination. We believe this framework is as close to identifying convergence on the same complementary geometries as can be achieved using unpaired data. Analysis methods that predict binding site residue similarity (e.g. clonotyping, or the novel paratyping (Richardson et al., bioRxiv 2020) and Ab-Ligity (Wong et al., bioRxiv 2020) tools from our group that do not condition on germline identity) could identify the Fvs with the maximal chance of being functionally equivalent. Alternatively, if looking for binders to a previously structurally characterised epitope, the genuine paratope residues could be derived and sequence similarity across these positions can be used to infer binding propensity.

We are currently processing all publicly available single cell sequencing data that captures BCRs for a new version of our Observed Antibody Space database (Kovaltsuk *et al*. 2018). We considered the following studies that have released paired sequencing data:

Goldstein *et al*. (2019) Massively parallel single-cell B-cell receptor sequencing enables rapid discovery of diverse antigen-reactive antibodies. *Communications Biology*
Setliff *et al*. (2019) High-Throughput Mapping of B Cell Receptor Sequences to Antigen Specificity. *Cell*
Horns *et al*. (2020) Memory B Cell Activation, Broad Anti-influenza Antibodies, and Bystander Activation Revealed by Single-Cell Transcriptomics. *Cell Reports*
Adamo *et al*. (2020) Myocardial B cells are a subset of circulating lymphocytes with delayed transit through the heart. *JCI Insight*
Eccles *et al*. (2020) T-bet+ Memory B Cells Link to Local Cross-Reactive IgG upon Human Rhinovirus Infection. *Cell Reports*
Alsoussi *et al*., (2020) A Potently Neutralizing Antibody Protects Mice against SARS-CoV-2 Infection. *Journal of Immunology*
King *et al*., (2020) Antibody repertoire and gene expression dynamics of diverse human B cell states during affinity maturation. *bioRxiv*
Chen *et al*., (2020) BCR selection and affinity maturation in Peyer's patch germinal centres. *Nature*

For the purposes of our study, we require longitudinal pre- and post- infection/vaccination data across several individuals, to contextualise the amount of structural sharing after vaccination. The closest dataset to our requirements is Horns *et al*. 2020. However, while they perform classic Illumina sequencing before and after vaccination, they only perform single-cell sequencing after vaccination, and only study a single individual, making it impossible to derive shared response structures. Coupled with this, we could only find processed CDRH3/CDRL3 pairings, not the full VH/VL reads, precluding Fv structural modelling.

We believe it is only a matter of time until an appropriate dataset is released, and fully agree with the editor that this would provide the ideal test case for Repertoire Structural Profiling. It should lead to a step change in accuracy and speed for the method, as artificial pairing is both time consuming and cannot ensure natural expressibility. While we alluded to the benefits of paired sequencing data in our introduction, we have redoubled this by adding a short paragraph to the discussion highlighting how Repertoire Structural Profiling would benefit from paired data.

To address the point that we cannot prove the same Fv structure appears in different individuals after vaccination, we have also reworded our manuscript title.

The following manuscript change was made:

>TITLE
Old: Evidence of Antibody Repertoire Functional Convergence through Public Baseline and Shared Response Structures
New: Public Baseline and Shared Response Structures Support the Theory of Antibody Repertoire Functional Commonality

>DISCUSSION
New: "Whilst ever we must artificially pair VH/VL sequencing datasets, we cannot conclusively prove that multiple individuals raised the same Fv in response to vaccination. This could soon be rectified with the advent of single-cell sequencing studies investigating vaccine response dynamics (Horns *et al*., 2020). Repertoire Structural Profiling could readily be applied to such data by skipping the combinatorial pairing step, which would be expected to improve both speed and accuracy."

Reviewer 1 Comments

Raybould et al. present a new approach to assess the degree to which antibodies with common modes of function exist across individuals in a population. The authors note that, to date, sequence-based analyses have not directly explained the broad, consistent immunogenic response to pathogens in a population, and suggest using structural comparisons to estimate functional commonalities that escape analyses based on sequence alone. Their approach first reduces the number of VH and VL sequences in an individual's sequenced repertoire by clustering sequences grouped by shared framework regions and with the same CDR lengths. Templates for each CDR are grafted onto the framework, and the VH–VL orientation was modelled by examining 20 predictive residues. Any sequences that could not be modelled by this process are not considered. After models are constructed, they are clustered to identify groups of similar structures. The sets of models from individuals' repertoires are then compared to identify structurally similar antibodies shared among individuals, termed public distinct structures. The authors find that approximately 3% of the structures are public, compared to 0.022% of sequences that can be classified as public through clonotype analysis. The authors then apply this analysis to a set of pre- and post-immunization repertoires to identify structurally similar antibody responses across three individuals.

This an interesting approach for exploring the frequency of groups of canonical CDRs and frameworks appearing together within and among individuals, but I do not believe the evidence, as reported, supports the authors' objective or specific claims. In consideration of this along with the contextualization of the method with respect to the other work in the field, I do not feel this work meets the high standards for publication in PLOS Computational Biology.

We regret that the reviewer does not believe that the evidence we have provided is strong enough to support our claims. We hope that our responses to their comments, the new analysis provided in this document, and the corresponding additions to the manuscript will allay any concerns about the paper's validity and suitability for PLOS Computational Biology.

In the abstract the authors state, "The antibody repertoires of different individuals ought to exhibit significant functional commonality, given that most pathogens trigger a successful immune response in most people. Sequence-based approaches have so far offered little evidence for this phenomenon. For example, a recent study estimated the number of shared ('public') antibody clonotypes in circulating baseline repertoires to be around 0.02% across ten unrelated individuals. However, to engage the same epitope, antibodies only require a similar binding site structure and

the presence of key paratope interactions, which can occur even when their sequences are dissimilar."

This raises a few questions that must be addressed:

(1) Why would one expect that individual immune responses would all target the same epitope? Indeed, polyclonal responses demonstrate that many epitopes can be the target of an immune response;

We agree with the reviewer that almost all antigens induce a polyclonal antibody response, usually against several different epitopes on the pathogen surface. In this paper, we do not require that all three individuals respond to all the same flu vaccine epitopes upon pathogenic exposure, but rather that a subset of 'immunodominant' epitopes are recognised across all three individuals. As per our discussion:
"Target-focused screening libraries against immunodominant epitopes are commonly derived through sequence analysis of longitudinal Ig-seq studies that track the immune response of many individuals to the same antigen."

The concept of immunodominant epitopes have been long-documented in diagnostics (see early work by Mordasini *et al*. (2006) *J. Clin. Microbiol.*) and vaccinology, and can been exploited to create simpler antigen immune stimulants that initiate a less polyclonal response (see this recent study on SARS-CoV-2 Mukherjee *et al.* (2020) *Vaccines*). We also note that:

1. Antigen surfaces with many characterised antibody co-crystal complexes can usually be clustered into discrete targeted regions with particular antibody binding orientations (e.g. SARS-CoV-2: Brouwer *et al*. Science 2020, Fig. 5; Barnes et al., bioRxiv 2020, Extended Data Fig. 10) In theory, an antigen surface is simply an electron cloud and could be bound in any way that can contribute enough interaction energy. However, that we observe discrete epitopes strongly implies that antibody binding sites are subject to topological restrictions that render some regions of the antigen surface intractable.

2. Coupled with this, certain epitope topologies are more likely than others to be more complementary to the spectrum of structures sampled in the baseline repertoire. This is most clearly seen in the inverse case: pathogens with epitopes that require long protruding CDRH3 loops for binding, such as HIV, usually lead to chronic illness as these topologies are rarely found in the naïve repertoire.

We have constructed our vaccination study in such a way that it is possible to derive the structures shared between just two individuals and not all three. With BCR-seq data from many individuals responding to the same vaccine, one could consider relaxing the restrictions such that post vaccination structures are considered convergent if they appear in at least N responding individuals, similar to how convergent clonotyping is usually performed (e.g. Galson *et al*. bioRxiv 2020). This relaxation may enable the identification of responding antibody structures to less immunodominant epitopes.

We have incorporated an additional reference to the assumption of immunodominance in the flu vaccination results section.

>RESULTS
Structurally Profiling a Flu Vaccine Response
New: "Often these expanded lineages are seen across many different individuals after vaccination,

implying particular pathogenic epitopes are ‘immunodominant’ — more susceptible to immune recognition (Mordasini *et al*. 2006, Mukherjee *et al*. 2020, Brouwer *et al*. 2020).”

(2) Clonotyping has a much higher threshold for labeling a sequence “public” because it is attempting to capture sequence convergence as opposed to epitope-binding or paratope convergence. While two distinct sequences can bind the same epitope while not being classified as public via clonotyping, two antibodies having similar backbone conformations (and thus would be classified as sharing a public structure) does not imply that they bind the same epitope or even the same antigen. How can comparisons of these concepts be made such that they reveal an immunological truth?;

We thank the reviewer for the opportunity to clarify the relationship between clonotyping and Repertoire Structural Profiling.

Clonotyping is fundamentally an attempt to capture convergent BCR maturation through whole CDRH3 sequence identity and closest V gene/J gene origins. It is highly useful for antibodies where VH (and particularly CDRH3) dominates antigen binding, and near-germline CDRH1/CDRH2 offer supporting complementary. However, scenarios where the above provisos are not met find clonotyping to be less reliable. Even in contexts more suited to clonotyping, it does not capture “immunological truth”. Crucially, CDRH3s of over 80% sequence identity can have markedly different CDRH3 structures (Fig 2B of Kovaltsuk et al. Frontiers in Immunology, 2018), and since similar binding site structure is a prerequisite of functional equivalence (to avoid clash and to hold residues within their interaction distance of the paratope) this precludes clonotyping from being able to prove any two VHs target the same epitope. V and J gene assignments can also be incorrect, particularly for sequences with high SHM counts, and thus offer no guarantee of identifying a genuine evolutionary maturation pathway.

Repertoire Structural Profiling also has a central flaw that precludes it from being able to categorise two antibodies as functionally convergent – in our case we do not consider their relative paratope interaction profiles. However, what it does ensure is the other key tenet of functional commonality: that the two antibodies have similar CDR backbone conformations. Interestingly, this can be achieved even if antibodies have very different global sequences (e.g. in the case of the CDRH3 loop, Fig 2A of Kovaltsuk et al. Frontiers in Immunology, 2018). Antibodies that can adopt the same structure need only possess a similar enough paratope residue profile to accommodate binding to the same epitope, meaning that within these structural sets are likely to be subsets of antibodies with different germline origin and more remote whole-CDRH3 sequence identity that can engage the same epitope. We argue this orthogonal approach is necessary because while convergent clonotypes can appear across individuals in response to antigen stimulation, barely any common clones exist even in very deep-sequenced naïve repertoires of the same individuals. Given that they likely responded to similar immunodominant epitopes (strongly suggested by the convergent post-vaccination clonotypes), they must have done it from different naïve clonotype starting points.

One possible way to capture these mappings could be to lower the CDRH3 sequence identity threshold below the field-benchmarked lower bound of 80%, pulling together more antibodies with CDRH1s & CDRH2s that map to the same V genes. However, this approach is flawed: you both lose confidence that clustered Fvs have similar interaction profiles and still have no knowledge as to whether the clustered CDRH3s can adopt backbone similar conformations.

Repertoire Structural Profiling completely relaxes the clustering criterion of CDRH3 residue sequence similarity and instead concentrates on clustering antibodies that are structurally similar across their entire binding site (both HCDRs and LCDRs). Further analysis methods that predict binding site

residue similarity (e.g. clonotyping or the novel paratyping (Richardson *et al*. bioRxiv 2020) and Ab-Ligity (Wong *et al*. bioRxiv 2020) tools from our group that do not condition on germline identity) are then needed to hone in on the Fvs with the maximal chance of being functionally equivalent across individuals. Alternatively, if looking for binders to a previously structurally characterised epitope, the actual paratope residues could be derived. Structural alignment to identify the closest Public Baseline representative followed by sequence identity/similarity across the actual paratope positions for each Fv could then be used to infer which Fvs may share a common binding mode.

We agree that we have not made it clear enough that antibodies adopting the same distinct structure are not necessarily functionally equivalent. We have therefore changed the title and made significant changes to the manuscript to rectify this.

The following manuscript changes were made:

>TITLE
Old: Evidence of Antibody Repertoire Functional Convergence through Public Baseline and Shared Response Structures
New: Public Baseline and Shared Response Structures Support the Theory of Antibody Repertoire Functional Commonality

>ABSTRACT
Old: “Here, we search for evidence of functional convergence in human antibody repertoires by comparing the antibody structures they contain.”
New: “Here, we search for evidence of geometric similarity/convergence in human antibody repertoires by comparing the antibody structures they contain.”

Old: “Our approach is the first computational method to provide support for the long-assumed levels of baseline repertoire functional commonality.”
New: “Our approach is the first computational method to find levels of BCR commonality commensurate with the pluripotency of the naive immune repertoire, and could therefore be applied to find more genetically distant antibodies with functional equivalence.”

>INTRODUCTION
Old: “However, clonotyping is only likely to identify a small subset of the true number of functionally equivalent antibodies. This is because it assumes that antibodies require a similar genetic background and high CDRH3 sequence identity to achieve complementarity to the same epitope. In reality, similar binding site structures and paratopes can be achieved from different genetic origins (Mitsunaga et al., 2019) and with surprisingly low CDRH3 sequence identity (Kovaltsuk et al., 2017). Conversely, false positives can be detected since high CDRH3 sequence identity and the same genetic origins can lead to markedly different binding site topologies (Kovaltsuk et al., 2017). It is also the case that not every epitope is naturally suited to CDRH3-dominated binding, instead preferring broader engagement by multiple CDRs (Dunbar *et al*., 2014).”
New: “However, clonotyping is only likely to identify a small subset of the true number of functionally equivalent antibodies. This is because it assumes that antibodies require a similar genetic background and high CDRH3 sequence identity to achieve complementarity to the same epitope. In reality, similar binding site structures and paratopes can be achieved from different genetic origins (Mitsunaga *et al*., 2019; Richardson *et al*., 2020) and with surprisingly low CDRH3 sequence identity (Kovaltsuk *et al*., 2017) (conversely, false positives can arise where antibodies with high CDRH3 sequence identity and the same genetic origins adopt markedly different binding site topologies (Kovaltsuk *et al*., 2017)). It is also the case that not every epitope is naturally suited to

CDRH3-dominated binding, instead preferring broader engagement by multiple CDRs (Dunbar *et al.*, 2014), putting less selection pressure on CDRH3 sequence identity."

Old: "It is difficult to capture these functionally equivalent antibodies by sequence alone. An alternative approach would be to compare the three-dimensional structures of the antibodies, as binders to a given epitope are likely to adopt a similar geometry with residues capable of recapitulating key binding interactions at equivalent topological locations."
New: "It is difficult to reliably identify these hidden functionally equivalent antibodies within a clonotyping framework, as simply reducing the sequence identity threshold value lowers confidence in paratope residue similarity and increases the risk of grouping antibodies with fundamentally different binding site topologies. An alternative approach to relaxing the clustering criterion would be to ignore CDRH3 residue similarity and instead to just group antibodies with similar three-dimensional structures, as binders to a given epitope are very likely to adopt a similar geometry. Geometrically-similar antibodies with sufficiently similar residue interaction profiles could then be capable of recapitulating key binding interactions at equivalent topological locations.

Old: "In this paper, we further refine this repertoire structural profiler, and apply the optimised pipeline to the task of repertoire functional screening."
New: "In this paper, we further refine this repertoire structural profiler, and apply it to cluster antibody repertoires based on predicted binding site topology"

Old: "Our data therefore represents the first computational evidence that sizeable functional commonality could exist in the baseline repertoires of different people."
New: "Our data therefore represents the first supporting computational evidence that considerably more functional commonality than suggested by clonotyping could exist in the baseline repertoires of different people."

>DISCUSSION
New: "Once grouped into public structures, Fvs can then be probed using an array of methods designed to measure binding residue similarity to identify the subset likely to have common functionality. For example, finding convergent clonotypes within the public baseline structures may bolster confidence in their functionally convergent role. Alternatively, methods that do not condition on predicted antibody genetic origin, such as paratyping (Richardson *et al.*, 2020) or Ab-Ligity (Wong *et al.*, 2020), could identify more genetically divergent antibodies capable of binding the same epitope. The public geometries themselves could also be harnessed in vaccinology, such as identifying an epitope targetable by a `Public Baseline' structure which may lead to a more reliable and convergent response."

(3) Why does having similar backbone conformations suggest that key paratope interactions are maintained? One can imagine two distinct antibodies (by essentially any definition) that could have nearly identical paratope–epitope interactions because those depend on the constellation of functional groups and hydrophobic surfaces arranged in space (i.e. it is possible to change the CDR loop lengths and conformations while maintaining the key interactions that give rise to an antibody's affinity and specificity).

We agree with the reviewer that having a similar backbone conformation does not guarantee that key paratope interactions are preserved. As we state in our discussion:

"Here we have shown that, at least from a structural perspective, there is considerable opportunity for functional commonality across the circulating resting-state repertoires of unrelated individuals (~3% of observed distinct structures are public across 10 individuals). The theoretical chemical

diversity that could be displayed on each of these scaffolds is large, so many of these grouped binding sites will not be complementary to the same antigen epitope. However, there is good reason to believe that a certain proportion are, as geometric similarity is a likely prerequisite of functional commonality, and our structural clustering approach offers a route to detecting and analysing these antibodies."

Finding an antibody with a similar binding site structure to a known binder ensures that putative paratope residues at the same position are close enough in distance to engage the epitope and can approach the epitope from a similar orientation. Thereafter, residues capable of engaging in the same intermolecular interactions may be functionally interchangeable. For example, a paratope hydrogen bond interaction between the hydroxyl group of a serine could be replaced with one to the hydroxyl of a threonine residue, if both are held at approximately equivalent topological positions.

The reviewer is correct that it is possible achieve similar geometries with loops of different length. We made the decision to stick with length-matched structural clusters as the RMSD comparisons are more rigorous between equal numbers of backbone atoms. We are encouraged by the number of public distinct structures we can identify despite this restriction, but acknowledge there will almost certainly be others we do not detect. The reviewer also discusses the edge case where two different antibodies could theoretically bind to roughly the same position on the antigen surface but using different CDRs to fulfil a similar interaction profile. Our method would not be able to identify these antibodies as functionally equivalent, but neither would any existing published method. The reviewer may be interested in our recently pre-printed 'Ab-Ligity' software, which describes paratopes as collections of interactions in 3D space without CDR dependence (Wong *et al*. bioRxiv 2020).

In response, we have added more to our discussion section describing the edge cases that our current method would fail to detect.

The following manuscript change was made:

>DISCUSSION
New: "We note that some edge cases, such as geometric similarity obtained using loops of different lengths, or antibodies that can use different CDRs to fit the same epitope via an alternative binding mode, are currently undetectable using our framework."

The authors argue that clonotyping is poorly suited to the task of identifying functionally similar antibodies "because it assumes that antibodies require a similar genetic background and high CDRH3 sequence identity to achieve complementarity to the same epitope. In reality, similar binding site structures and paratopes can be achieved from different genetic origins (21) and with surprisingly low CDRH3 sequence identity (22)." However, they do not provide evidence that the structure-based approach employed in this study would identify genetically diverse antibodies that are functionally similar across individuals. Unfortunately, I do not believe this structure-based approach can capture the kind of functional convergence required to shed light on the phenomenon under study.

We thank the reviewer for this point; analysis of the Fvs assigned to the same public structure should certainly have been included in the original manuscript. We have now supplied a case study of a Public Baseline structure mapped to all the Fvs predicted to adopt it. We demonstrate that while some of these Fvs derive from the same clonotype at the 80% sequence identity threshold, most are genetically diverse antibodies that could not be identified by a clonotyping framework. These grouped antibodies will not all bind to the same antigen epitope, however we believe

experimental validation would show it contains additional binders to the same epitope that were untraceable by clonotyping. We direct the reviewer to our reply to Reviewer 2 Major Comment 3, and particularly Tables R6 and R7.

Reviewer 2 Comments

Raybould and colleagues describe an alternative approach to defining shared public immune repertoires at the structural level to address the shortcomings of defining repertoires, their diversity, and their shared commonality by sequence. To achieve this, the authors classify 41 available NGS datasets of immune repertoires into clusters using their custom structural profiling pipeline which can predict structural classification based on certain features present in antibodies. They then model these representative antibodies from each cluster into Antibody Model Libraries which they assert is a better metric for representative antibodies in the immune repertoire than the classic sequence based clonotype definition. Additionally, the authors show that this method of classifying antibodies by structure can be applied to pre/post vaccination immune repertoire profiling to show an increase in overlapping antibodies upon common antigen challenge. In this case, a flu vaccine.

Significance -

This study is elegant in concept and execution. Currently the Ig-seq community at large has trouble reconciling the fact that the immune system is able to converge on epitopes given any common antigen challenge but we don't find much in way of shared sequences during large scale immune profiling experiments of the naive population. A simple way to reconcile would be to cluster by structure rather than sequence, but so far, a robust solution has yet to be presented. Here, Raybould and colleagues present a complex but feasible solution to this problem and this structural repertoire profiler may become a standard part of immune repertoire analysis in the community.

We thank the reviewer for their very kind words and for highlighting the role that Repertoire Structural Profiling and related structural methods could play in future immune repertoire analysis.

Major Concerns -

While I would like to see these major problems addressed, I don't necessarily think they preclude publication.

1. You use Briney et al extremely low percentage of shared repertoire as an impetus to this study. I would like to see at least an example in your clusters showing me why the Briney et al definition of clonotype would have missed groupings of clonotypes in this study. That is, what is the sequence divergence in one of these clusters? Is it a matter of just tuning a knob on sequence based clonotype definition to achieve the same clustering you arrive at in this study? Or would no amount of tuning to a sequence based clonotype definition allow me to achieve the same clustering methods as the structural profiler shown here. It seems you do some sequence clustering at 90% so perhaps just tuning the clonotype pairing at 90% would suffice.

We thank the reviewer for this point and think this will make a significant addition to the manuscript. We have have carried out this analysis and describe it below in our response to Major Comment 3, where we elaborate a Public Baseline structure with all the Fv sequences predicted to adopt it, and clonotype the VH sequences at different threshold values.

2. While you use Briney et al as a standard, you don't mention Soto et al 2019 which used more robust clustering methods to define clonotype. Using an 80% sequence similarity in HCDR3 in

addition to the classic V-J pairing, they found a shared repertoire of around 6% which jives more closely with the overlap presented here. This goes back to 1. Would tuning the knob capture the clustering you see here?

We thank the reviewer for bringing the study by Soto et al to our attention. We note that they analysed three individuals and found 6% public clonotypes shared between two of the individuals, 1% sharing in the other two-individual comparisons, and just 0.3% sharing across all three individuals, using their more lenient V3J 80% CDRH3 sequence identity clonotypes. This more lenient clonotype definition is unable to capture what must be a larger true underlying functional similarity between the three repertoires. We have elaborated this point in our response to Major Comment 3, where the Fvs predicted to adopt two public distinct structures are clonotyped using a more lenient CDRH3 sequence identity to show that "tuning the knob" within the clonotyping framework is unable to pool together most of the cluster members and potentially misses a subset that could bind to the same epitope.

Separately, we have also repeated the clonotyping of the naïve baseline dataset with the more lenient clonotyping method (Table R5). As expected, this shows a larger number of shared clonotypes across the Gidoni baseline repertoire samples, however this number is still very small (0.011% across ten individuals).

Dataset	Public Clonotypes (Briney Definition)	Public Clonotypes (Soto Definition)
Reference Clonotypes (S64)	70255 (100%)	68672 (100%)
Shared with S57	11 (0.02%)	1648 (1.30%)
And S5	0	358 (0.14%)
And S56	0	194 (0.04%)
And S83	0	78 (< 0.01%)
And S67	0	49 (< 0.01%)
And S84	0	23 (< 0.01%)
And S76	0	18 (< 0.01%)
And S54	0	11 (< 0.01%)
And S89	0	8 (< 0.01%)

Table R5: Tracking the number of public clonotypes shared across all datasets analysed up to that point. i.e. 358 clonotypes are present in S64, S57, and S5 according to the Soto V3J definition.

Simply clonotyping with the threshold set below 80% to recapitulate the degree of "sharing" seen between individuals by Repertoire Structural Profiling would not be a productive strategy. As the CDRH3 identity is lowered, you would increasingly lose confidence that the "clonal" antibodies have similar binding residue profiles (the primary advantage of clonotyping) and increase the risk of changing the CDRH3 conformation which could alone destroy binding. A combination approach with Repertoire Structural Profiling could be more productive, where first you derive Public Structures with confidence in preserving binding site geometries, and then could consider tuning down the clonotyping knob to identify plausible functionally similar VHs within the Public Structure cluster. RSP can also be used in conjunction with standard ≥80% sequence ID clonotyping to add confidence that two VH sequences in the same clonotype contribute to similar binding site geometries (see Table R7). We have added a discussion on the potential future relationships between Repertoire Structural Profiling and clonotyping to the manuscript.

The following manuscript changes were made:

>INTRODUCTION
"Such sequence-based approaches have contributed significantly to our knowledge of core immunology. For example, to estimate the true level of sequence similarity that exists across individuals, Briney et al. performed deep sequencing and clonotyping of the circulating baseline VH repertoires of ten volunteers (Briney *et al*., 2019). They found that just 0.022% of observed clonotypes were `public' (seen in everyone) and a similar study by Soto et al. found just ~1% of clonotypes were public across three unrelated individuals (Soto *et al*., 2019)."

>RESULTS
`Public Baseline' Structures in Unrelated Individuals.
"Clonotyping our baseline snapshots, even at the lower 80% CDRH3 sequence identity threshold used by Soto et al. (Soto *et al*., 2019), revealed < 0.01% public clones after five individuals (SI Table 4)."

>DISCUSSION
"Once grouped into public structures, Fvs can then be probed using an array of methods designed to measure binding residue similarity to identify the subset likely to have common functionality. For example, finding convergent clonotypes within the public baseline structures may bolster confidence in their functionally convergent role. Alternatively, methods that do not condition on predicted antibody genetic origin, such as paratyping (Richardson *et al*., 2020) or Ab-Ligity (Wong *et al*., 2020), could identify more genetically divergent antibodies capable of binding the same epitope. The public geometries themselves could also be harnessed in vaccinology - for example, identifying an epitope targetable by a `Public Baseline' structure may lead to a more reliable and convergent response."

>METHODS
Clonotyping. Clonotyping was performed to group antibodies with the same closest V and J gene, and either identical CDRH3 sequences, as in Briney *et al*. (Briney et al., 2019), or CDRH3 sequences within 80% sequence identity, as in Soto *et al*. (Soto *et al*., 2019).

>Table R5 has been added as SI Table 4.

3. Show me a model, some of its members, and the sequences they contain. I just want one represented model in the main figures to see exactly what one of these AML looks like. Particularly the CDR loops with different sequences but are predicted to have the same structure.

We thank the reviewer for this suggestion. We have added a characterisation of all the Fv sequences expected to adopt H14012+L14649, the closest Public Baseline structure to Ustekinumab. An alignment of H14012+L14649 to the Ustekinumab crystal structure is shown in Figure R3(A). This shows the majority of the 0.6Å deviation can be explained by a slightly different CDRH3 loop conformation. A total of 4911 Fv sequence pairings across the ten individuals were assigned to this Public Baseline structure, representing 155 unique VH sequences. Knowing the structure to be geometrically close to Ustekinumab, we then searched for the most similar of the 155 VH sequences, as evidence that similar CDR residue profiles can also be detected even when "searching by structure". Figure R3(B) shows the closest VH sequence, with 25/37 (68%) identical HCDR residues to Ustekinumab. Several mismatches are to similar residue types (e.g. S <-> T, Y <-> F). The CDRH3 is 66% identical, meaning clonotyping even at the 80% threshold would not pool the Ustekinumab VH and the AML VH sequence together.

A

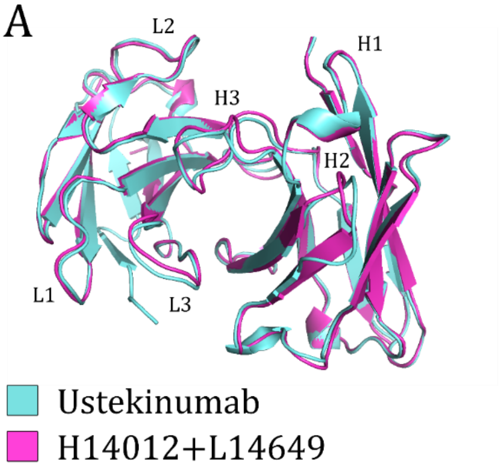

B

Ustekinumab, Gidoni

VH: EVQLVQSGAEVKKPGESLKISC**KGSGYSFTTYWLG**WVRQMPGKGLDW**IGIMSPVDSDIR**YSPSFQGQV
VH: -VQLVQSGAEVKTRGESLKLSC**KGSGYSFTSYWTG**GVRQMPGKGLEW**TGIIYPGDSDTS**NSPSFQGQV

TMSVDKSITTAYLQWNSLKASDTAMYYC**ARRRPGQGYFDF**WGQGTLVTVSS
TISADKSSSTGYLQWSSLKASDTAMYYC**ARREVGMGYFDY**WGQGTLVTVSS

VL: DIQMTQSPSSLSASVGDRVTITC**RASQGISSWLA**WYQQKPEKAPKSLI**YAASSLQS**GVPSRFSGSGSG
VL: DIQLSQSPSFLSASVGDRVTITC**RASQGISSSLA**WYQQKQGKAPKVLI**YAASTLQS**GVPTRFRGSGSG

TDFTLTISSLQPEDFATYYC**QQYNIYPYT**FGQGTKLEIK
TEFTLTISSLQPEDFATYYC**QQLNSCPPT**FGQGTRLEIK

C

VL: DIQMTQSPSSLSASVGDRVTITC**RASQGISSWLA**WYQQKPEKAPKSLI**YAASSLQS**GVPSRFSGSGSG
VL: EIVMTQSPATLSVSPGARATLSC**RASQSVNSNLA**WYQQKPGQAPRLII**FGSSTRST**GIPVRFSGGGSG

TDFTLTISSLQPEDFATYYC**QQYNIYPYT**FGQGTKLEIK
TEFTLTISGLQSEDFEVYYC**QQYNNWPYT**FGQGTKLEIK

Figure R3. (**A**) Alignment of the solved Ustekinumab crystal structure (3hmw) and the closest Public Baseline AML structure (H14012+L14649). (**B**) Comparison of the Ustekinumab Fv sequence and a Gidoni *et al*. naïve Fv sequence assigned by Repertoire Structural Profiling to the H14012+L14649 Public Baseline structure. The North-defined CDR regions of each chain are highlighted in bold. While both the Ustekinumab and Gidoni VH sequences align closest to the same V and J genes (IGHV5-51/IGHJ4), the CDRH3 sequences are only 66% sequence identical, and so would not have been assigned to the same VH clonotype. (**C**) An alternative VL sequence coupled to the same Gidoni VH sequence. This sequence has a more sequence similar CDRL3 but a less similar CDRL2.

We then surveyed the light chains associated with this VH and found two VLs (Figure R3B & Figure R3C) with similar motifs to Ustekinumab's VL. Figure R3B's VL is more similar across the three CDRs (22/26, 85%), while Figure R3C's is closer in the CDRL3 but less identical in the CDRL2. Both VLs also derive from different IGKV germlines to the Ustekinumab VL (Ustekinumab: IGKV1D-16, R3B VL: IGHV1-9, R3C VL: IGKV3-15). Overall, the Fv described in Figure R3B is 75% sequence identical to Ustekinumab across all 6 CDRs.

Overall, this expands our original observation that Public Baseline structures are geometrically close to therapeutically exploitable antibodies with an example that they can be close in interaction profile too. This strengthens our case for using Repertoire Structural Profiling/Public Structures to design general antibody screening libraries.

We then investigated the H14012+L14649 cluster itself, looking at the diversity of genes and CDRH3s represented within. We performed clonotyping (80% seqID threshold) on the 155 sequence non-redundant VH chains to see how many different heavy chain clonotypes were mapped to the H14012+L14649 Public Baseline structure.

The 155 VH sequences clustered into 141 clonotypes, whose germline gene combinations are shown in Table R6 (assigned by ANARCI Dunbar et al. Bioinformatics 2016). As clonotyping conditions on antibodies having the same V and J gene identities, these different combinations would not never be pooled into a single category by clonotyping.

V Gene	J Gene	Count
V5-51	J4	83
V5-51	J3	18
V5-51	J6	3
V5-51	J5	2
V1-8	J4	9
V-1-8	J6	3
V5-10-1	J4	8
V5-10-1	J5	1
V1-2	J4	7
V1-2	J5	1
V1-3	J4	4
V1-46	J3	1
V1-69-2	J4	1

Table R6: HV and HJ germline gene combinations seen across the 155 VH sequences mapped to the H14012+L14649 Public Baseline structure. By far the most abundant lineage is IGHV5-51/IGHJ4 (same as Ustekinumab).

Twelve clonotypes had multiple occupancy (Table R7). Of these, 3 were found across different individuals:

Clonotype V5-51+ARPYGSGSYSDY+J4 was seen in S64, S54, and S76
Clonotype V5-51+ARQGYGDYVTDY+J4 was seen in S67 and S76
Clonotype V5-51+ARMGARPGYFDY+J4 was seen in S89 and S76

The Briney et al. clonotype definition would have missed these three inter-individual overlaps, but the Soto definition is able to capture them. So there is an improved overlap between Repertoire Structural Profiling and clonotyping when “tuning the knob” down to a lower (80%) threshold value. However, RSP predicts that a geometrically similar binding site can exist in all ten individuals. Methods that can predict paratope similarity across all six CDRs may be able to find considerably more antibodies within each distinct structure cluster with similar enough interaction profiles to be functionally equivalent.

V Gene	J Gene	CDRH3 (Individual)	Overall Occupancy
V5-51	J4	**ARPYGSGSYSDY** (S64)<br>ARHDGSGSYSDY (S54)<br>ARQYVSGSYSDY (S76)	3
V5-51	J4	**ARQGYGDYVTDY** (S67)<br>ARQDYGDYVVDY (S76)	3
V5-51	J6	**ARLGCYYYGMDV** (S5)<br>ARQGYYYYGMDV (S5)	2
V5-51	J4	**ARQSSNWNGVDY** (S89)<br>ARQSSNWNGGDY (S89)	2
V1-69-2	J4	**ATSRDGYNNLDY** (S5)<br>ATARDGYNKLDY (S5)	2
V5-51	J4	**AGQDDYGDYVDY** (S89)<br>ARQGDYGDYVDY (S89)	2
V5-51	J4	**AVGGGWYAVGDY** (S5)<br>AVGGGWYGGGDY (S5)	2
V5-51	J4	**ARMGARPGYFDY** (S89)<br>ARPGEDGLEFDY (S76)	2
V5-51	J4	**AREETIARASDY** (S76)	2
V5-51	J4	**ARPDYAPSGIDY** (S64)	2
V5-51	J5	**ARLKKKENWFDP** (S67)	2
V5-51	J4	**ARRPMSYPEFDY** (S67)	2

Table R7: Clonotypes of multiple occupancy amongst the 155 VH chains mapped to the H14012+L14649 Public Baseline structure. The bold CDRH3 is the cluster representative.

The following manuscript changes were made:

>RESULTS
Proximity to Therapeutics
To show that similar binding site residue profiles can also be found by Repertoire Structural Profiling, we examined `Public Baseline' distinct structure `H14012+L14649' as a case study (Fig. 3).

This structure lies within 0.64 of the therapeutic Ustekinumab (SI Table 7). Examining the backbone-aligned structures shows this difference lies in slightly different CDR loop structures assigned to the CDRH2, CDRH3, and CDRL3 loops (Fig. 3A). We then examined all 4,911 Fv sequences assigned to this distinct structure across the ten individuals (S64 through S89), looking for the closest CDR sequence identity matches to Ustekinumab. The most similar of the 155 sequence-unique VH sequences assigned to this distinct structure is shown in Fig. 3B. While both the Ustekinumab and `Public Baseline' VH sequences most closely aligned to the same V and J genes (IGHV5-51/IGHJ4), the CDRH3 sequences are only 66% sequence identical, and so would not have been assigned to the same VH clonotype (the typical minimum threshold is 80\% as used in Soto *et al*. (Soto Nature 2019). This VH was coupled both with the VL sequence shown in Fig. 3B and with a VL sequence shown in Fig. 3C. The VL in Fig. 3B VL is more identical across the three CDRs (22/26, 85%), while the one in Figure 3C is closer in CDRL3 identity but considerably less so in CDRL2 identity. Both these VLs derive from different IGKV germlines to the Ustekinumab VL (Ustekinumab: IGKV1D-16, Fig. 3B VL: IGHV1-9, Fig. 3C VL: IGKV3-15). Overall, the Fv described in Fig. 3B is 75% sequence identical to Ustekinumab across all 6 CDRs.

This level of sequence and structural similarity between clinical-stage therapeutic antibodies and representatives of the `Public Baseline' repertoire suggests that Repertoire Structural Profiling could prove an effective tool for designing general screening libraries containing promising drug leads.

VH Sequence Profiling the `H14012+L14649' Distinct Structure

We performed clonotyping (80% sequence identity threshold (Soto *et al.* 2019*)* on the 155 sequence non-redundant VH chains to determine the diversity of heavy chain clonotypes mapped to the `H14012+L14649' Public Baseline structure. The VH sequences clustered into 141 distinct clonotypes, whose germline gene combinations as assigned by ANARCI (Dunbar *et al*., 2016) are shown in SI Table 8. As clonotyping conditions on antibodies having the same V and J gene identities, it would never pool these VHs into a single category. Twelve of the 141 clonotypes have multiple occupancy (SI Table 9). Three were found across multiple individuals:

V5-51+ARPYGSGSYSDY+J4: seen in S64, S54, and S76
V5-51+ARQGYGDYVTDY+J4: seen in S67 and S76
V5-51+ARMGARPGYFDY+J4: seen in S89 and S76

This shows how Repertoire Structural Profiling can be used in conjunction with clonotyping to bolster confidence in the functionally equivalent role of convergent clones. Novel methods currently being developed that can predict paratope similarity across all six CDRs (Richardson *et al*. 2020; Wong *et al*. 2020) may be able to find considerably more antibodies within each distinct structure cluster with similar enough interaction profiles to be functionally equivalent. To facilitate future investigations into this area, we supply the Fv sequences across all ten individuals assigned to each `Public Baseline' distinct structure as Dataset 3.

> Figure R3 was added as the new Figure 3.
>Tables R6 and R7 were added as SI Table 8 and SI Table 9, respectively

Minor Concerns -

1. How well does your modellable FVs overlap with the VH-VL pairing frequencies seen in papers like Dekovsky et al which, while not as deep as a single heavy chain NGS experiment, does provide a decent snapshot of paring frequencies in the human repertoire?

We thank the reviewer for this question. To test this, we have evaluated the heavy-light V family pairing frequencies across the Public Baseline structures (sequences from S64) and compared it to the pairings given in DeKosky et al. (Figure R4).

Our Baseline Structures cover broadly the same diversity of V families as DeKosky et al. (65 combinations represented in DeKosky, compared to 63 combinations in our set). We also find similar trends across the usage profiles (to the resolution of within a neighbouring 5% bin), with notable enrichment seen for similar VH-VL pairings across the IGHV1 and IGHV3 families. Some light chain families are unrepresented in our structures: IGKV5 and IGLV4, although their abundances are <1% per family pairing in the DeKosky set.

Our VH-VL pairing step compares each possible Fv sequence pairing to solved Fv structures from the PDB, and given how highly engineered it is, there is no guarantee that “modellable” pairings would mimic natural pairing frequencies seen in Ig-seq data. We were encouraged to discover through the reviewer’s suggested experiment that our Antibody Model Library contains V family pairings that are broadly consistent with a natural repertoire.

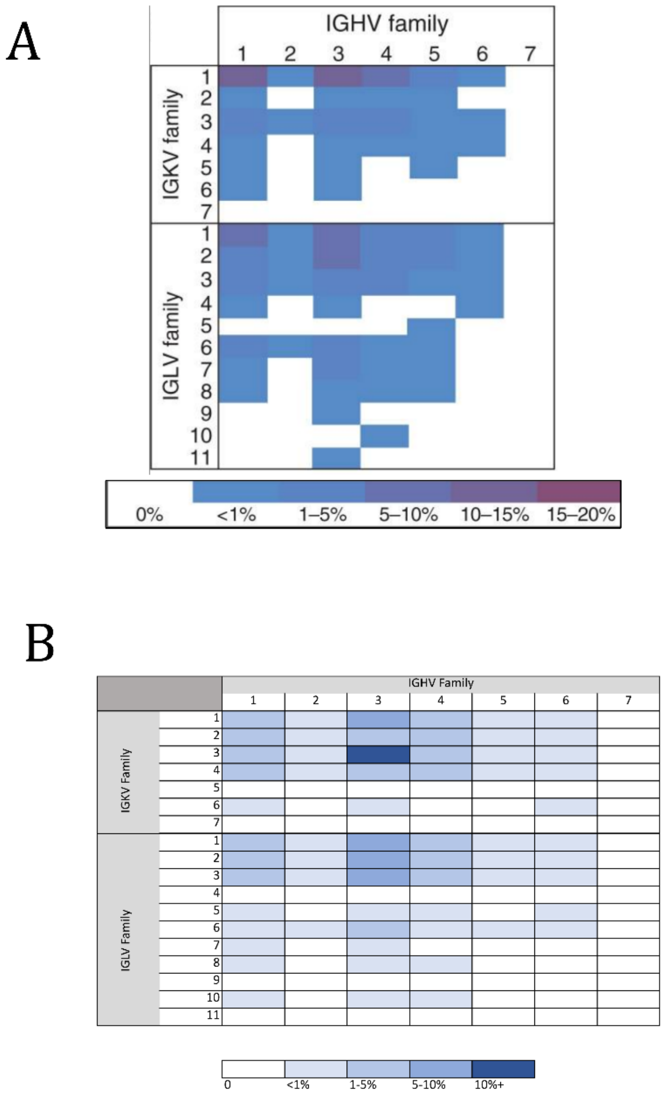

Figure R4. (A) Reproduced from DeKosky et al. Nat Biotechnol 2013 (Figure 2). (B) The equivalent V family heat map for the Public Baseline structures (sequences from S64).

We have added reference to this analysis to the main manuscript and have added the accompanying figure to the Supporting Information.

The following manuscript change was made:

>RESULTS
Characterising the Public Baseline Structures
“Finally, we considered the constituent paired V genes across the `Public Baseline' structures. As our pairing algorithm only predicts modellable Fv pairings based on PDB structures, we compared our IGHV/IG[K/L]V pairing frequencies with those observed in DeKosky *et al*.'s study of *c*. 2000 natively-paired antibodies (Figure S7). Our `Public Baseline' gene pairing frequencies were very similar to DeKosky *et al*.'s native sample, with the IGHV1/IGKV1-4, IGHV1/IGLV1-3, IGHV3/IGKV1, IGHV3/IGKV3, and IGHV3/IGLV1-4 pairings the most abundant.”

2. This analysis is extremely complex and I would love to see a protocol capture, in this paper, or a separate publication showing a user how they could potentially adopt this pipeline to their own work. Without that, it's really hard to check the methodology

We thank the reviewer for this point. We package our codebase that is relevant to therapeutic antibody discovery/development into a virtual machine (SAbBox), which is available under a free academic or paid industrial license. After publication, this code is earmarked to be included in the next version of SAbBox and could therefore be directly adopted into drug discovery pipelines or be privately developed for in-house industrial or academic purposes.

3. It's my understanding that sequence-based clustering after vaccination also predicts a lot of shared clonotypes. Can you compare your method of a 17% shared antibody by structure to a sequence based approach?

We thank the reviewer for this suggestion. We have run V3J clonotyping over the 'Before Vaccination' and 'After Vaccination' VH sequences according to both the Briney and Soto definitions.

Dataset	Public Clonotypes, Briney Def'n (% Public)	Public Clonotypes, Soto Def'n (% Public)
V1 Before (Reference Clonotypes)	48,459 [100%]	43,166 [100%]
Shared with V2 Before	310 [0.32%]	262 [0.34%]
And V3 Before	38 [0.03%]	32 [0.03%]
V1 After (Reference Clonotypes)	86,837 [100%]	75,927 [100%]
Shared with V2 After	444 [0.27%]	1,508 [1.02%]
And V3 After	33 [0.02%]	272 [0.13%]

Table R8. The number of public clonotypes shared across V1+V2 and V1+V2+V3 before and after vaccination. The central column uses the Briney *et al*. clonotyping method (100% CDRH3 sequence identity), while the final column uses the Soto *et al*. clonotyping method (80% CDRH3 sequence identity).

In summary, according to the Soto clonotyping method (80% threshold), **0.03**% of clonotypes are shared between all individuals before vacation, and this increases to **0.13**% of clonotypes shared after vaccination. This compares with **17.8**% shared binding site geometries before vaccination and **19.2**% shared binding site geometries after vaccination.

The following manuscript changes were made:

>RESULTS
Structurally Profiling a Flu Vaccine Response
"Again, a significant number of public distinct structures were observed in `V1',`V2', and `V3' (`Public Before Vaccination' structures, 17.78% (236,792/1,444,597) of all `Before Vaccination' distinct structures). This indicates that the identification of `Public Baseline' structures in the previous section was unlikely due to serendipitous Ig-seq amplification bias. Interestingly, 17.78% is a similar percentage of sharing as that seen after three baseline snapshots (16.12%; 71,743/445,045). <u>For context, the proportion of all clonotypes that were public before vaccination was just 0.03% (Soto *et al*. definition, SI Table 8).</u>

The degree of structural sharing appears to increase after vaccination, with 19.23% (350,710/1,823,648) public structures across the three volunteers. This is consistent with a degree of repertoire structural convergence driven by exposure to the same pathogenic epitopes <u>and with an increase in the proportion of public clonotypes after vaccination to 0.13% (SI Table 8).</u>

> Table R8 was added as SI Table 8

4. When you use CD-HIT to cluster by sequence at 90%, are there still more than one model for each cluster? Or does this does not collapse the entire structural cluster into a single sequence? It would be great to see an itermediate diversity of just heavy chain pairings after this step and a few select repertoire overlaps.

We thank the reviewer for this point. Almost certainly there are instances where we collapse antibodies with similar global VH sequences into a single sequence even though the component CDRs may have been assigned to different template structures. Unfortunately, this sequence clustering is crucial to the computational tractability of the pairing protocol, which currently involves billions of considerations of potential VH/VL pairings, and scales prohibitively for around 10x the number of VH and VL chains to be considered. Our hope is that in time more paired sequencing data will be released in the public domain, rendering the pairing step redundant. Analysis would start from the same point (consideration of VH and VL CDR modellability) and then proceed directly to orientation assignment and structural clustering based on templates, without need for sequence clustering.

Reviewer 3 Comments

The work by Raubould et al is very interesting and important in highlighting the importance of structural knowledge of the antibody repertoire functional commonality.
The work is very well performed and interesting to read.

We thank the reviewer for their kind words.

I have one major query that I think can help in validating the message further and better understand the role of the modelling in the final outcome.
Could this extent of 'public' (shared) antibodies be the artefact of the modelling - i.e. only could consider the modellable part of the repertoire (with templates etc.) which impose some restrictions on the CDRs and hence you would naturally find some recurring patterns of the CDRs shared across different individuals?
In other words how can one be sure that the modelling methods do not introduce bias that will increase the "public" sequences?

We thank the reviewer making this very relevant point. As we state in the discussion, we can only make claims about the modellable portion of these repertoires and as more structures become modellable this could impact upon the proportion of `public’ structures.

Our consideration of Random Repertoires shows that the theoretical binding site space that could be modelled with the existing FREAD templates is around 10x greater than that sampled by the real repertoires, which we consider an indication that the central narrative of this paper (that a sizeable set of commonly-observed public structures exists across multiple individuals) is unlikely to change upon solving more structures.

However, through characterisation of the S64 public baseline sequences and the S64 private baseline sequences (those mapped to a non-public distinct structure), we observed that an inflated proportion of public structures at higher CDRH3 loop lengths (see Figure R1A), which we believe can largely be accounted for by modellability.

There exists a paucity of CDRH3 loop structural templates for both short and long CDRH3s. For shorter CDRH3s this is not too problematic – structurally, the closure constraint means there are fewer geometrically feasible backbone conformations, and the coverage we have should be

sufficient to capture this diversity – instead the reason we see few public baseline structures with short CDRH3s (lengths 5-8) is the low abundance of short CDRH3s in the BCR-seq data.

However, the structural space available to long CDRH3 (20+) loops is enormous, and we have nowhere near full structural coverage. As a result, if an Fv containing a long CDRH3 loop is considered modellable, it is more likely to be assigned to a structural template further away from its true structure, thus artificially overinflating the numbers of long CDRH3s that look structurally similar. This can be seen in the noticeably higher proportion of public structures than private structures with longer CDRH3 loops. Therefore, as more templates of longer CDRH3 loops emerge and CDRH3 modellability and model accuracy improves, we might reasonably expect a drop in the number of long-H3 public baseline structures.

For more moderately-sized CDRH3 loops (5-19), the proportions of public and private structures are much closer – *i.e.* if all you were told was that an antibody has a length 5-19 CDRH3, you could not infer from that alone that the structure was public or private. We therefore have confidence that public assignments for these structures are not unduly influenced by modellability. However, we have now added to the manuscript mention that some of the public structure assignments (particularly those with CDRH3 lengths >= 22) should be approached with caution.

The following manuscript changes were made:

>RESULTS
Characterising the `Public Baseline' Structures
<u>CDR3 Length Usages</u>
"We compared the CDRH3, CDRL3 and CDRH3+CDRL3 distributions of the S64 Fv sequences assigned to a `Public Baseline' structure against those assigned to a `Private Baseline' structure. The CDRL3 and CDRH3+CDRL3 length usages demonstrate that `Public Baseline' structures are not an artefact of using shorter CDR3 loops with more limited conformations. In fact, we find that modellability bias is likely to be overstating the proportion of `Public Baseline' distinct structures with longer CDRH3 loop lengths (**SI Fig. 5**). The structural space available to long CDRH3 (20+) loops is enormous, and we have relatively poor structural coverage. As a result, if an Fv containing a long CDRH3 loop is considered modellable, it is more likely to be assigned to a structural template further away from its true structure, thus artificially inflating the numbers of long CDRH3s that look structurally similar. These longer CDR length `Public Baseline' structures should therefore be treated with caution and, as more templates of longer CDRH3 loops emerge improving CDRH3 modellability, we would expect their numbers to decrease to the public:private ratios seen at more moderate CDRH3 lengths."

>DISCUSSION
"This might suggest that a large portion of structural commonality will remain across the (as yet) unmodellable regions of structural space, <u>although we do expect the number of `Public Baseline' structures with long CDRH3 loops to fall, as modellability may be increasing this figure</u>."

>Figure R1 was added as SI Figure 5

Also it is interesting to know which specific features of the antibody distinct structure elements contribute most to the public response structures and specifically are contributing to the observed 3% distinct structures in common between the studied individuals' repertoire.

We thank the reviewer for this suggestion. In response we have added two new downloads to the resources page of our website (http://opig.stats.ox.ac.uk/resources):
(a) all Fv sequences predicted to lie on each public baseline distinct structure ("Dataset 3")
(b) all FREAD CDR templates assigned to each public baseline distinct structure ("Dataset 1")

These new datasets allow for the sequences and/or structures of any public baseline distinct structure of interest to be interrogated. As an initial investigation, we report the median number of different FREAD CDR templates assigned to each `Public Baseline' distinct structure (Table R9). As expected, CDRH3 conformation appears to dominate in defining a distinct structure.

CDR	Median Number of FREAD Templates/`Public Baseline' Structure
H1	3
H2	4
H3	2
L1	5
L2	8
L3	7

Table R9. The median number of FREAD templates for each CDR represented in a given Public Baseline structure.

We also note that:

1. The Antibody Model Library derived from the S64 sequences predicted to adopt public baseline distinct structures remains available to download from our group's website http://opig.stats.ox.ac.uk/resources

2. In reply to Editor Comment B, we have performed a comparative characterisation of the S64 sequences assigned to the public baseline distinct structures against the S64 sequences assigned to other distinct structures. This includes the above-discussed CDRH3 length distribution plot, as well as HV/LV gene sequence identity profiles (Figure R1B), showing that proximity to germline is not a good indicator of public vs private structure.

The following manuscript changes were made:

>RESULTS
Characterising the `Public Baseline' Structures
CDR Template Usages
We investigated the number of different structural templates that were assigned to each CDR in a `Public Baseline' distinct structure (SI Table 6). As expected, the lowest median number of different templates per distinct structure was recorded for the CDRH3 loop (2 templates/structure), consistent with the large structural variation within the region driving the definition of distinct binding site structures. Collectively, the light chain CDRs recorded considerably more FREAD templates per structure (median of 20 templates/structure) than the heavy chain CDRs (median of 9 templates/structure). We have supplied the sets of FREAD templates assigned to each CDR of each distinct structure to facilitate further structural characterisations of distinct structures of interest (Dataset 1).

VH Sequence Profiling the `H14012+L14649' Distinct Structure
To facilitate future investigations into this area, we supply the Fv sequences across all ten individuals assigned to each `Public Baseline' distinct structure as Dataset 3.

>Table R9 was added as SI Table 6
>Datasets 1 and 3 were added to *http://opig.stats.ox.ac.uk/resources*

Reviewer 4 Comments

The paper "Evidence of Antibody Repertoire Functional Convergence through Public Baseline and Shared Response Structures" studies the structural similarity of antibody from repertoires of different individuals, with the aim of identifying public structures, and to study the progression of immune responses.
The aim is important, but I have some concerns on some of the methods. In general, I believe that the paper should tone down its results and discuss the bottlenecks and critical points of the analysis

We thank the reviewer for their analysis of our manuscript. While we believe our findings are of high relevance to the field, we can see how some sections of our original manuscript could have been misinterpreted to infer that all antibodies with the same binding site structures are functionally equivalent. We also acknowledge that, as we artificially pair VH and VL data, we cannot prove that the same Fv structures are simultaneously present in different individuals. We have therefore made significant changes to clarify the interpretation of public structures (see manuscript changes made in reply to Reviewer 1, Major Comment 2), and have also made the title of the manuscript more accurate.

The following manuscript changes were made:

>A set of amendments to clarify the role of Repertoire Structural Profiling and the implication of public distinct structures (as these are extensive, please see Reviewer 1, Major Comment 2 for changes)

>TITLE
Old: Evidence of Antibody Repertoire Functional Convergence through Public Baseline and Shared Response Structures
New: Public Baseline and Shared Response Structures Support the Theory of Antibody Repertoire Functional Commonality

>DISCUSSION
New: "Whilst ever we must artificially pair VH/VL sequencing datasets, we cannot conclusively prove that multiple individuals raised the same Fv in response to vaccination. This could soon be rectified with the advent of single-cell sequencing studies investigating vaccine response dynamics (Horns *et al.*, 2020). Repertoire Structural Profiling could readily be applied to such data by skipping the combinatorial pairing step, which would be expected to improve both speed and accuracy."

Major
1. All the analyses are performed on bulk sequencing data, where VH and VL sequences are not paired, by pairing all VHs to all VLs. This means that most of the antibody studied are, in the best

case, potentially observable but not present in the repertoire, and in the worst case, impossible to be produced.

We thank the reviewer for this very valid point and, in response, we made the two manuscript changes described above.

As an example following the paper: long H3 and L3 are rarely seen together. This means, for example, that pairing short H3 with short L3 antibodies would generate a subset of structures with limited diversity, but that are not necessarily real. This point has to be discussed and addressed. For example: is there a bias in the CDR length in public structures with respect of non public ones?

We thank the reviewer for the opportunity to address this point. We have plotted the CDRH3, CDRL3, and CDRH3+CDRL3 length distributions over the S64 sequences assigned to `Public Baseline' structures *vs*. the S64 sequences assigned to `Private Baseline' structures (Figure R1). We found broadly similar distributions across all three properties, and no evidence that "short H3-short L3" combinations were enriched in the public data, creating an artificial degree of structural sharing. We suspect this is due to the very low abundance of short CDRH3 sequences in human antibody repertoires (the CDRH3 sequence lengths in this baseline sample by Gidoni *et al* are consistent with other datasets explored in Raybould et al., 2019, *PNAS*; Kovaltsuk *et al*., 2020, *Plos Comp Bio*).

We saw an increase in the public:private structure ratio as CDR length increased, a phenomenon we attribute to modellability (expanded upon in our reply to Major Comment 3 below).

The following manuscript changes were made:

>RESULTS
Characterising the `Public Baseline' Structures
CDR3 Length Usages
"We compared the CDRH3, CDRL3 and CDRH3+CDRL3 distributions of the S64 Fv sequences assigned to a `Public Baseline' structure against those assigned to a `Private Baseline' structure. The CDRL3 and CDRH3+CDRL3 length usages demonstrate that `Public Baseline' structures are not an artefact of using shorter CDR3 loops with more limited conformations. In fact, we find that modellability bias is likely to be overstating the proportion of `Public Baseline' distinct structures with longer CDRH3 loop lengths (**SI Fig. 5**). The structural space available to long CDRH3 (20+) loops is enormous, and we have relatively poor structural coverage. As a result, if an Fv containing a long CDRH3 loop is considered modellable, it is more likely to be assigned to a structural template further away from its true structure, thus artificially inflating the numbers of long CDRH3s that look structurally similar. These longer CDR length `Public Baseline' structures should therefore be treated with caution and, as more templates of longer CDRH3 loops emerge improving CDRH3 modellability, we would expect their numbers to decrease to the public:private ratios seen at more moderate CDRH3 lengths."

>Figure R1 has been added as SI Figure 5.

2. The paper claims that the antibody structural space is far from a random selection, and that this hints that structural similarity might be fundamental to identify antigenic pressure in a better way than one can obtain by performing sequence-based analyses. While this makes sense, I believe that the randomisation tests performed fail to prove so. All the randomisation tests are performed by uniform sampling from a large pool of potential models with reasonable CDR3 lengths, coming from all possible germlines, both human and murine. So, the tests performed say that the structures in the repertoire are not generated by randomly sampling such a diverse dataset, and this is correct,

bot not overly interesting. The repertoire of each individual is only generated by a non uniform selection from a small pool of genes (and only human of course). Moreover, many structures in the template database might be heavily engineered or contain a large amount of somatic mutations, so that they will be quite diverse from the probably nearly naive repertoires.
The authors should perform additional randomisation tests to show that there is a structural selection and that this might be due to antigenic pressure. as a first step, the randomised dataset could be generated by having the same distribution of CDR lengths and the same structural templates used in the actual repertoires.

We thank the reviewer for the suggestion to only use templates that have previously been assigned by FREAD to a human CDR sequence in building the Random Repertoires (RRs). This change definitely improves confidence in our Random Repertoires, as it elegantly accounts both for FREAD assignment biases and gene-imposed structural restrictions. As the reviewer suggests, many PDB structures are highly engineered, and the single "organism" tag applied to each one is at best approximate and at worse arbitrary (e.g. in the case of chimerics). The set of templates assigned by FREAD to each human sequence have the species origins shown in Table R2; the proportion of "human" origin templates has been upweighted, suggesting a degree of correction for true species conformation bias. We note that the proportion of "mouse origin" templates remains significant – we consider this reflects both that a proportion of binding site geometries are accessible to both species and that PDB species assignments have low reliability.

We designed Random Repertoires to be reflections of the totality of structural space available to the human antibodies given the sequences sampled in the real repertoire. As length is a crucial parameter determining which binding site structures are accessible, we were careful to only allow Random Repertoires access to the same combinations of CDR lengths that were seen in each real repertoire. This criterion should be sufficient to capture the maximum structural diversity theoretically accessible to each real repertoire.

Using this new setup, we rebuilt all RR benchmarks and recalculated the overlap values. As the results of this investigation are extensive, we redirect the review to our reply to Editor Comment A, which steps through each of the ramifications of the change.

In summary, as expected, the new restrictions on CDR template usage reduced the structural space sampled by the RRs and increased the expected overlap between real repertoires. However, the expected overlap still decays rapidly to < 0.01 % public structures by the fifth individual (compared to 8.06% public structures observed across the 5 real repertoires). Our conclusions are unchanged and are bolstered by the more rigorous way of generating the random baseline.

Manuscript Changes: Extensive, please see reply to Editor Comment A for details

They could also generate artificial sequences (e.g. with IgOR, see "High-throughput immune repertoire analysis with IGoR"). If shared structures are observed in such artificial repertoires also, this would mean that these are due to the genetic set-up of Igs, and not to antigenic pressure

We thank the reviewer for this suggestion. Our experience with synthetic immune repertoire sequence generators including IgoR has been that they tend to output a high proportion of structurally non-viable sequences (e.g. missing the crucial canonical C23-C104 disulfide bridge), and so we do not have confidence that the current technologies have captured natural antibody "syntax" sufficiently to provide a structurally-relevant random 'natural sequence' baseline. We believe the new Random Repertoires, incorporating the reviewer's suggested restriction on FREAD template

usage, provide evidence that the genetic set-up of Igs is not responsible for the observed structural convergence. We also note that our VH and VK/VL germline sequence identity investigation in reply to Editor Comment B (Figure R2) shows that sequences adopting public structures lie no closer on average to their 'parent' V gene than those adopting private structures.

3. The amount of "modellable" Igs is just a small fraction of all the sequences, specifically those for which proper templates are available. This means that the high level of similarity might be caused by a selection bias, in conjunction with similarity by proxy, meaning that limited number of templates will force similarity for sequence modeled on the same templates.
The first problem is hard to addressed, but I would suggest discussing it. For example, the 3% public structures might become negligible if all possible antibodies from the repertoire are considered, and not only the ones for which modeling is possible.

We thank the reviewer for this point. We tried to make this clear in our original discussion section:

"All of the structural analysis in this paper is limited to the antibody chains that are currently predicted to be modellable, and so there remain regions of natural structural space uninvestigated. Nevertheless, we show that antibody repertoires tend only to explore highly focused regions of currently-modellable structural space (~10% of the diversity expected if templates were explored randomly across the same combinations of CDR lengths).

But we have now developed this paragraph further to incorporate the reviewer's comment that it is possible that this will result in a dilution in the proportion of public structures.

Through an investigation of CDRH3 length distributions across the `Public' and `Private' structures, we saw a surprising enrichment for the assignment of CDR structures with longer CDRH3 loop lengths as public. We attribute this to modellability, as the structural space available to long CDRH3 (20+) loops is enormous, and we have nowhere near full structural coverage. As a result, if an Fv containing a long CDRH3 loop is considered modellable, it is more likely to be assigned to a structural template further away from its true structure, thus artificially inflating the numbers of long CDRH3s that look structurally similar. Therefore, as more templates of longer CDRH3 loops emerge and CDRH3 modellability and model accuracy improves, we might reasonably expect a drop in the number of long-H3 public baseline structures. We have therefore included discussion on this point to the manuscript.

The following manuscript changes were made:

>RESULTS
Characterising the `Public Baseline' Structures
<u>CDR3 Length Usages</u>
"We compared the CDRH3, CDRL3 and CDRH3+CDRL3 distributions of the S64 Fv sequences assigned to a `Public Baseline' structure against those assigned to a `Private Baseline' structure. The CDRL3 and CDRH3+CDRL3 length usages demonstrate that `Public Baseline' structures are not an artefact of using shorter CDR3 loops with more limited conformations. In fact, we find that modellability bias is likely to be overstating the proportion of `Public Baseline' distinct structures with longer CDRH3 loop lengths (**SI Fig. 5**). The structural space available to long CDRH3 (20+) loops is enormous, and we have relatively poor structural coverage. As a result, if an Fv containing a long CDRH3 loop is considered modellable, it is more likely to be assigned to a structural template further away from its true structure, thus artificially inflating the numbers of long CDRH3s that look structurally similar.

These longer CDR length `Public Baseline' structures should therefore be treated with caution and, as more templates of longer CDRH3 loops emerge improving CDRH3 modellability, we would expect their numbers to decrease to the public:private ratios seen at more moderate CDRH3 lengths."

>DISCUSSION
All of the structural analysis in this paper is limited to the antibody chains that are currently predicted to be modellable. This means there remain significant regions of natural structural space uninvestigated and once these become characterisable, the currently observed proportion of public structures may become diluted. Nevertheless, we show that antibody repertoires tend only to explore highly focused regions of currently-modellable structural space (~10% of the diversity expected if templates were explored randomly across the same combinations of CDR lengths). This might suggest that a large portion of structural commonality will remain across the (as yet) unmodellable regions of structural space, although we do expect the number of `Public Baseline' structures with long CDRH3 loops to fall, as modellability may be increasing this figure."

As for the second problem, some template subsampling could give an idea of the effect of template paucity on the estimated diversity.

We thank the reviewer for this suggestion. To estimate the impact of a year's worth of missing templates on estimated diversity, we removed all predicted modellable Fvs from S64/S57 with at least one orientation/CDR assigned to a PDB released between January 2018 and February 2019, the database timestamp (a total of 276 PDB entries, a database reduction of -7.94%). We note that this is the strictest potential impact of template paucity, as some of these CDRs may have been reasonably modelled by other pre-2018 templates.

This reduced dataset S64 from 6,420,211 to 5,301,993 modellable Fvs (-17.4%)
This reduced dataset S57 from 7,225,630 to 6,241,183 modellable Fvs (-13.6%)
As expected, this had the effect of reducing the numbers of measurable distinct structures observed in the S64 and S57 datasets (Table R10). The reduction lay at around 10%; that this figure is smaller than the percentage reduction of modellable Fvs shows that a significant portion of the structural space explored by the 2018-2019 templates was already explored in the pre-2018 templates.

Dataset	Distinct Structures (All FREAD Templates Available)	Distinct Structures (Only pre-2018 templates available)
S64	209,394	186,677 (-10.8%)
S57	201,039	179,763 (-10.6%)

Table R10: Comparing the original estimated structural diversity figures for S64/S57 with estimated structural diversity if predicted modellable Fvs with templates assigned to PDB structures from 2018/2019 were removed.

Finally, we looked at what influence this reduction in measurable structural diversity had of the proportion of public distinct structures. Interestingly, the proportion of public structures remained high, and within 1% of the original value (Table R11). A higher proportion of public structures was detected with increased modellability, which suggests that future increases in coverage may not necessarily dilute the proportion of public structures to an insignificant value.

# Repertoires (DS Added)	Cumulative Public and Private Distinct Structures (value with all templates)	Public Distinct Structures; % Public (values with all templates)
1 (S64)	186,677 (209,394)	186,677; 100% (209,394; 100%)
2 (S57)	305,948 (340,915)	87,920; **28.7**% (100,824; **29.57%)**

Table R11: Assessing the impact on % public structures of removing modellable Fvs with templates assigned to PDB structures from 2018/2019 (values in brackets are without removing 2018/2019 templates).

The following manuscript changes were made:

>RESULTS
`Public Baseline' Structures in Unrelated Individuals
Finally, we tested whether the observed proportion of `Public Baseline' structures would have been significantly different if the experiment had been run on an earlier database. We repeated Repertoire Structural Profiling for the two most structurally diverse datasets S64 and S57 removing any modellable Fv pairing whose best predicted template for any region was released by the PDB in 2018 or later. As expected, the number of predicted modellable Fv distinct structures in each sample fell from 209,394 and 201,039 to 186,677 and 179,763 respectively (a fall of around 10%). We then performed structural overlap analysis on these sets of distinct structures, finding a total of 305,948 distinct structures across both datasets, of which 87,920 were public to both S64 and S57. This degree of structural sharing (28.7%) is comparable to the degree observed with access to the entire FREAD database (29.6%).

Minor
Introduction:
"V and J gene transcript origins and above a certain percentage (same length) Complementarity-Determining Region H3 (CDRH3) sequence identity." -> the (same length) bit is unclear at this point, so remove it or expand it

We agree with the reviewer and have removed the parenthesis.

Results
"41 Gidoni volunteers with sufficiently deep reads (see Methods)." -> with sufficient sequencing depth

We agree that this is a better wording and have changed it as suggested.

Methods
- "Sequence clustering. The modellable chains were then sequence clustered using CD-HIT (39) at a 90% sequence identity threshold, to reduce the number of VH-VL pairing comparisons to a computationally-tractable number."
I strongly advice against using CD-HIT for this kind of analyses. CD-HIT only assures that sequencing in the same cluster are >90% identical (even not so, since it doesnt properly take indels into consideration), but in large dataset, it will retain many cases where sequences that are >90% are in different clusters. This means that, the larger the initial dataset, the more likely 2 random representatives might actually be similar in sequence

We thank the reviewer for their advice and agree that CD-HIT is not the optimal way of sequence clustering any sequence dataset. However, it fulfils the role we need it to play in our algorithm, namely quickly and sizeably reducing the sequence space and thus computational complexity for the pairing step, which as it currently stands involves billions of comparisons. We manually inspected the first couple of datasets and were content with the clustering that CD-HIT was providing, though as the reviewer suggests there were edge cases. Ultimately, we anticipate that sequence identity clustering will be rendered unnecessary once appropriate pairing sequencing data is made publicly available, as the computational complexity of artificial pairing will no longer be a consideration.

--

Yours sincerely,
Matthew Raybould
