# Supplementary material for: Public Baseline and shared response structures support the theory of antibody repertoire functional commonality
Source: PLoS Comput Biol. 2021 Mar 1;17(3):e1008781. doi: 10.1371/journal.pcbi.1008781 (PMC7951972; doi:10.1371/journal.pcbi.1008781)
Supplement: Attachment — Submitted filename: PCB_SecondRound_ReplytoReviewers.pdf [file pcbi.1008781.s021.pdf]

Dear Professor Dunbrack,

Thank you for your positive recommendation and for your recognition of our efforts to address the reviewer concerns. We completely understand the reservations of reviewer 1 and agree that this study should be followed-up with structural analysis of paired sequencing samples, once these sequencing methodologies achieve sufficient depth to yield representative samples of entire repertoires. Nonetheless, we hold that the antibody repertoire structural biases identified by Repertoire Structural Profiling on unpaired data are significant and warrant further investigation and characterisation. We trust that future papers will build upon these foundations to establish the true extent of functional commonality across BCR/antibody repertoires.

We have responded to the remaining concerns of Reviewer 1 below.

--

Reviewer 1

The reworked version of the manuscript offers additional analyses, clarifies many points raised during the initial review and more precisely describes many of the claims offered by the available evidence. I would also like to acknowledge the immense amount of work that went into not only the original version of the manuscript but also in preparing these revisions. In my initial review of this work, I felt that the claims being made were too sweeping; the adjustments made by the authors have resulted in a much more nuanced and clear report. However, the addition of this nuance and clarity reveals that the central claim – that analyzing antibodies in terms of structure of the Fv instead of only considering shared genetic lineage among antibodies will yield a larger set of antibodies identified as similar to one another – is essentially tautologically true. Coupling that with the various sources of modeling errors and the inability to determine which of the structurally similar antibody models actually target the same epitopes leads me to feel that this work does not meet the high standards for publication in PLOS Computational Biology.

We thank the reviewer for their extensive consideration of the manuscript and for acknowledging the benefit of our additional analyses. We regret that despite these amendments they still do not consider the work to meet the standards for publication in PLOS Computational Biology. Like the reviewer, we were not surprised to observe a greater number of antibodies with similar structures than the number of antibodies belonging to the same clonotype when comparing two individuals' naïve repertoires (though we note that the overlap is statistically significant compared to random, unlike findings from sequence-based naïve repertoire overlap analysis). The more impactful finding of this analysis is that so many of the same binding site topologies were predicted across 10+ individuals' antibody repertoires, a "naïve structural basis set", which implies antigenic selection and could contribute to why certain epitopes are so immunodominant. Clustering antibody repertoires in this way also leads to new possibilities in bioengineering and repertoire analysis. Firstly, Repertoire Structural Profiling (RSP) allows for general natural immunoglobulin screening library design (*in vitro* or *in silico*) that covers the diversity of predicted public naïve structural space. Secondly, RSP allows mappings to be drawn between particular binding site topologies of interest (either a solved antibody-antigen complex close to a 'public structure', or a convergent topology observed after infection/vaccination) and more genetically diverse antibody starting points with the same predicted topology that could be intrinsically and/or rationally engineered to become co-complimentary (*i.e.* antibody scaffold hopping). This knowledge could be used to design antigen-targeted or even epitope-targeted screening libraries around these lead sequences. Finally, it offers orthogonal information to

clonotyping/other sequence-based tools for functional annotation. RSP could therefore be used to build confidence that two antibodies belonging to the same clonotype contribute to topologically similar binding sites, or in combination with more lenient clonotyping that sets as lower threshold for required CDRH3 sequence identity so long as the broader binding site topology is preserved.

Minor textual changes were made to the abstract, results, and discussion section to clarify the potential applications of Repertoire Structural Profiling and to emphasise the possible link between observed epitope immunodominance and public structures (see Tracked Changes file: “PCB_Manuscript_TrackedChanges.pdf”).

1) One question that came to mind as I read this paper is the extent to which the computationally-expensive step of generating structural models provides a benefit over less expensive sequence-based approaches. If different-length CDR loops that could lead to similar paratopes (a task that is made extremely challenging by the inability to produce pairs of heavy and light chains that are known to form the donor’s functional antibodies) aren’t being considered, could an analysis following a sequence-based assignment to canonical CDR cluster types and the same 20-residue orientation prediction provide the same information as a complete structural model? The observation that longer CDR 3 loops lead to an overestimate of the degree of similarity between antibodies suggests considering only the length of the CDR loops can lead to template selection-based modeling errors that may be present elsewhere (and possibly inflating the number of public structures).

We thank the reviewer for this question. To clarify, full Fv structural models are only built after identifying relevant topologies by template clustering. Instead, independent FREAD predictions of each HCDR or LCDR’s best structural template are used during the structural clustering step.

Sequence-based classification of canonical CDR loops (e.g. using our tool SCALOP, 10.1093/bioinformatics/bty877, which uses the CDR sequence alone to predict canonical loop structures) would represent an alternative to FREAD’s predictions for identifying structurally similar CDRH1-2 and CDRL1-3 loops, but would not offer a route to identifying similar CDRH3 loop structures as they are non-canonical given our current levels of data. As each CDR is modelled by FREAD within ABodyBuilder during AML creation, we chose to implement FREAD throughout the entire RSP pipeline to ensure as much consistency as possible in prediction.

As the reviewer states, a limitation of FREAD is that it will only predict templates of the same length as the CDR sequence input, unlike SCALOP which can assign similar structures that span up to two CDR lengths (though these only exist in CDRL1 [L1-16-17-A] and CDRL3 [L3-9,10-A and L3-10,11-A] based on the current levels of data). We note that very common CDR length-variant similar structures could be identified at the end of the pipeline by performing CDR length-independent distance analysis over the final set of homology models. This could implicate a group of public ‘distinct structures’ that are topologically similar but that differ in the length of at least one of their CDRs. One could then consider merging these two distinct structures into a single category for subsequent analysis.

A deeper question is “whether different-length antibodies can lead to similar paratopes”. E.g. if the loop in question isn’t contributing any binding interactions, why can’t antibodies with

shorter versions of that loop not also bind? But due to the dependence on the antibody-antigen interaction profile, this would have to be considered on an epitope-by-epitope basis.

The factors leading to the difficulty of modelling longer CDRH3 loops accurately are related to systematic biases resulting in a paucity of longer data, rather than the fact that we are limited only to considering same-length templates. For example, longer CDRH3 loops are rare in humans and mice (the two dominant species in the PDB), longer CDRH3 loops have a wider conformational space accessible to them, and longer CDRH3 loops are more likely to be flexible and so be more challenging to crystallise.

We have amended the discussion to include the potential for identifying common CDR length variable but similar distinct structures through consideration of the homology models in the Antibody Model Library (AML).

Discussion
"We note that some edge cases remain in our analysis. It may be possible to identify structurally similar binding sites that use loops of different lengths through analysis of the resulting AMLs, but they are not readily detectable during this implementation of the clustering protocol. Antibodies that can use different CDRs to fit the same epitope *via* an alternative binding mode are also currently undetectable using our framework."

2) In the response to reviewers, the authors present a compelling case for ignoring the organism assignment from the PDB, but the case for combining template structures across organisms (with the possible exception of CDR H3) remains thin. While the antibody structures in the PDB are heavily engineered in such a way as to make the organism assignment uninstructive, the source of the frameworks and each CDR can be identified separately (e.g. with an HMM) and then grouped by source organism as opposed to PDB organism label. Does this procedure produce better results?

We thank the reviewer for their suggestion. While it would be an interesting future investigation to see whether different forms of template segmentation could improve homology-based antibody modelling algorithms, developing a machine learning or HMM classifier to assign each PDB-derived CDR sequence to a probable species origin is beyond the scope of this manuscript. As per our previous set of replies, we have seen no evidence so far that this would help improve model accuracy (e.g. we know that restricting by PDB-labelled origin does not currently help). Engineering is also often performed to deliberately change the properties from a protein belonging to the "parent" species to that of a new organism (e.g. humanisation). On top of this, we know through analysis of our Observed Antibody Space database that identical sequence CDRs can be observed across species, throwing into question the maximum achievable accuracy of any classification approach.

3) In the proximity to therapeutics section, the authors state "Of the 66 therapeutics with known structures that had at least one antibody in our 'Public Baseline AML' with 6 identical CDR lengths, all had a structural partner in the AML within a Cα Fv RMSD of 1.84Å…" Similar to the above concerns, how does this value change if one performs a sequence-based assignment to canonical clusters instead of only considering the length? While I would expect the number of antibodies under consideration to decrease, I would expect the maximal RMSD to decease as well (which would suggest that building structural models are not providing a benefit over a more robust sequence-based analysis).

Aside from the aforementioned issue of not being able to predict a canonical form for the CDRH3 loop, our work on length-independent canonical forms (10.1093/bioinformatics/bty877) indicates that only two loops currently possess at least one canonical class spanning multiple lengths: CDRL1 (L1-16-17-A), and CDRL3 (L3-9,10-A; L3-10,11-A). The reviewer's suggested amendment may therefore reduce the influence of the light chain CDR length on the number of distinct structures detected, however as most of the variable signal driving structural clustering resides the HCDRs (and mostly in CDRH3 conformation, see Table S6), we would not expect this methodological change to significantly alter the results.

4) In the same section, it is unclear how RSPs would aid in the design for screening libraries – I would recommend adding a description of the value they would add over in in addition to widely used antibody campaigns and how the major limitations of those techniques (including immunogenicity) would be effectively reduced. For example, when building a phage display library as suggested in the discussion, each mutation toward binding a particular target would essentially reset the question of immunogenicity and would require complete in vivo exploration for confirmation, thus neutralizing the advantage of developing the library from AMLs.

We thank the reviewer for this point. RSP analysis provides a continuum of naturally-expressed VH+VL sequences expected to sit upon each public topology in the AML. AML sequences ought therefore to represent low immunogenicity starting points, certainly lower than libraries built upon random CDR mutations from a set of human germline sequences (as per the discussion section: "We hypothesise that human `Public Baseline' structures are more likely to display low levels of human immunogenicity and be versatile binders"). No optimisation of these initial AML compounds for immunogenicity should be necessary.

While historically considered in series (*i.e*. first achieve good affinity, then fix the developability), it is now the scientific consensus that productive antibody maturation platforms should consider affinity and developability issues in tandem. Starting from these human sequences, we would therefore suggest an informatics-led approach built from an analysis of large datasets of human antibodies. Mutations to the sequence should be targeted and only allowed between residues seen in the same context in human sequences. While this does not guarantee a "zero immunogenicity" antibody, it should certainly reduce the risk.

We have added reference to this approach to affinity maturation in the discussion:

Discussion: "To chemically elaborate this `Public Baseline' structural basis set, a phage display library on the order of $10^6$-$10^7$ sequence-unique human antibodies could be created from the many different Fv sequences predicted to adopt each public distinct structure. Mutations are likely required to optimise the affinity of a `Public Baseline' antibody against a chosen epitope. If performed randomly, these mutations could negate the benefits of using natural antibody leads. However, tools such as "Hu-mAb" can distinguish human sequences from those of other organisms to extremely high accuracy (cit.). Integrating these algorithms into affinity maturation pipelines to restrict mutations to those that do not decrease sequence "humanness" should help to preserve the low immunogenicity of `Public Baseline' lead antibodies."

5) I appreciate the adjustment of the title of this paper. However, upon further consideration

of the uncertainty in VH/VL pairing, the limitation of which sequences are considered modellable, the revealed biases associated with template availability and selection, and the uncertainty of the utility of the structural models over other sequence-based approaches (i.e. canonical cluster assignment and comparison), I feel it is too strong. While I agree with the overall thrust of this paper – that thinking in terms of structure of the Fv will almost certainly suggest a larger degree of functional commonality compared to only considering shared genetic lineage among antibodies – the previously listed limitations to generating models with atomic accuracy severely complicates the degree to which support for a particular theory can be extracted from them. I would recommend further adjusting the title to "Public Baseline and Shared Response Structures are Consistent with the Theory of Antibody Repertoire Functional Commonality".

We thank the reviewer for this suggestion. We consider the wordings "support" and "are consistent with" to convey a very similar message and would prefer to stick with our first adjusted title for brevity.

Reviewer #2: none

We thank the reviewer for their first-round contributions to the manuscript.

Reviewer #3: I am satisfied with the response to my queries, I think the authors have made a substantial effort in addressing all the criticisms.

We thank the reviewer for their kind words and for their first-round contributions to the manuscript.

--

In addition to these changes, as the public AMLs were created using template databases dating back to February 2019, we will be releasing two updated public AMLs (with database timestamps of November 2020) alongside the manuscript. We are still supplying the original AMLs, as they were the basis for the analysis described throughout the manuscript, but recommend that researchers use the two new datasets with improved structural coverage. These will soon be available from Zenodo and the "Resources" page of our website.

Yours sincerely,
Matthew Raybould
